# Comparison of 24-Month Outcomes After Treatment for Distal Radius Fracture: The WRIST Randomized Clinical Trial

**DOI:** 10.1001/jamanetworkopen.2021.12710

**Published:** 2021-06-17

**Authors:** Kevin C. Chung, Hyungjin Myra Kim, Sunitha Malay, Melissa J. Shauver

**Affiliations:** 1 Section of Plastic Surgery, Department of Surgery, University of Michigan Medical School, Ann Arbor; 2 Michigan Medicine Comprehensive Hand Center, University of Michigan, Ann Arbor; 3 Center for Statistical Consulting and Research, University of Michigan, Ann Arbor

## Abstract

**Importance:**

Distal radius fractures (DRFs) are common injuries among older adults and can result in substantial disability. Current evidence regarding long-term outcomes in older adults is scarce.

**Objective:**

To compare outcomes across treatment groups at 24 months among adults with DRFs who participated in the WRIST trial.

**Design, Setting, and Participants:**

The Wrist and Radius Injury Surgical Trial (WRIST) randomized, international, multicenter trial was conducted from April 1, 2012, through December 31, 2016. Participants were adults aged 60 years or older with isolated, unstable DRFs at 24 health systems in the US, Canada, and Singapore. Data analysis was performed from March 2019 to March 2021.

**Interventions:**

Participants were randomized to open reduction and volar locking plate system (VLPS), external fixation with or without supplementary pinning (EFP), and percutaneous pinning (CRPP). The remaining participants chose closed reduction and casting.

**Main Outcomes and Measures:**

The primary outcome was the 24-month Michigan Hand Outcomes Questionnaire (MHQ) summary score. Secondary outcomes were scores on the MHQ subdomains hand strength and wrist motion.

**Results:**

A total of 304 adults were recruited for the study, and 187 were randomized to undergo surgery, 65 to VLPS, 64 to EFP, and 58 to CRPP; 117 participants opted for closed reduction and casting. Assessments were completed at 24 months for 182 participants (160 women [87.9%]; mean [SD] age, 70.1 [8.5] years). Mean MHQ summary scores at 24 months were 88 (95% CI, 83-92) for VLPS, 83 (95% CI, 78-88) for EFP, 85 (95% CI, 79-90) for CRPP, and 85 (95% CI, 79-90) for casting, with no clinically meaningful difference across groups after adjusting for covariates (χ^2^_3_ = 1.44; *P* = .70). Pain scores also did not differ across groups at 24 months (χ^2^_3_ = 2.64; *P* = .45). MHQ summary scores changed from 82 (95% CI, 80-85) to 85 (95% CI, 83-88) (*P* = .12) between 12 and 24 months across groups. The rate of malunion was higher in the casting group (26 participants [59.1%]) than in the other groups (4 participants [8.0%] for VLPS, 8 participants [17.0%] for EFP, and 4 participants [9.8%] for CRPP; χ^2^_3_ = 43.6; *P* < .001), but malunion was not associated with the 24-month outcome difference across groups.

**Conclusions and Relevance:**

The study did not find clinically meaningful patient-reported outcome differences 24 months after injury across treatment groups, with little change between 12 and 24 months. These findings suggest that long-term outcomes need not necessarily be considered in deciding between treatment options. Patient needs and recovery goals that fit to relative risks and benefits of each treatment type will be more valuable in treatment decision-making.

**Trial Registration:**

ClinicalTrials.gov Identifier: NCT01589692

## Key Points

**Question:**

For patients aged 60 years and older with unstable distal radius fractures, are there 24-month outcome differences across the 4 treatment strategies of volar locking plates, external fixation, percutaneous pinning, and casting?

**Findings:**

In this randomized clinical trial of 182 adults from 24 health systems, there were no significant differences in any 24-month outcome by treatment.

**Meaning:**

These findings suggest that there are no differences in 24-month outcomes based on treatment and that patients with unstable distal radius fractures treated with casting can experience good outcomes despite malunion, while avoiding the risks of surgery.

## Teaser

This randomized clinical trial compares 24-month outcomes among older adults with distal radius fractures across 4 treatment groups: volar locking plates, external fixation (with or without pinning), percutaneous pinning, and casting.

## Introduction

More than 85 000 Medicare beneficiaries sustain distal radius fracture (DRF) annually.^1^ Despite more than 200 years of experience in treating this injury since it was first described by Colles^2^ in 1812, the American Academy of Orthopaedic Surgeons guidelines published in 2009 indicated that there is insufficient evidence to justify any particular DRF treatment over another for older adults.^3^ The lack of evidence for comparative efficacy stems from the difficulty in conducting clinical trials when the treatment options are so disparate that recruiting a sufficient sample size can be difficult. This is particularly true for older adults, who resist being randomized and may experience barriers to follow-up such as lack of transportation.^4,5,6,7^

Literature is sparse regarding long-term, posttreatment DRF outcomes in older populations. Few studies followed older adults longer than 12 months, and those that did only reported results from the final assessment; intermediate outcomes to track the trajectory of recovery were missing.^8,9,10,11,12,13,14,15,16^ Long-term outcomes on injuries in older adults are especially germane because today’s older generation is living longer and leading more active lives than previous generations. In this era of value-based care and shared decision-making, long-term outcomes help to guide surgeons, patients, and their families through understanding the benefits and risks of each treatment.

To derive level 1 evidence for DRF treatment, we conducted the Wrist and Radius Injury Surgical Trial (WRIST). WRIST compared the 4 most commonly used treatments for DRF: open reduction and internal fixation with a volar locking plate system (VLPS), closed reduction and external fixation with a bridging fixator with or without supplemental k-wire fixation (EFP), closed reduction and k-wire fixation with percutaneous pinning (CRPP), and closed reduction and casting. WRIST has previously reported no clinically meaningful differences in hand outcomes by treatment groups at 12-month follow-up.^17^ The primary aim of this analysis is to examine 24-month outcomes of patients aged 60 years and older with DRF as a long-term outcomes aim stipulated by the National Institutes of Health. We hypothesized that 24-month outcomes would differ by the 4 treatment groups and that patients with malunion would have worse outcomes than those without malunion.

## Methods

 The WRIST protocol (Supplement 1) was approved by the institutional review board at the coordinating center and at all sites. A Data Safety and Monitoring Board appointed by the National Institutes of Health oversaw the study aims and conduct. Participants provided written informed consent. This study adheres to the Consolidated Standards of Reporting Trials (CONSORT) reporting guideline.

Patients with DRF who were aged 60 years or older and community-dwelling were screened for eligibility at 24 health systems located in Canada, Singapore, and the US from April 1, 2012, through December 31, 2016. Participants in the surgical (randomized) and casting (observational) groups had identical eligibility criteria: isolated fractures (concomitant ulnar styloid fracture was allowed) with displacement warranting surgical intervention (Arbeitsgemeinschaft für Osteosynthesefragen type A2, A3, C1, or C2 and meeting 1 of the following radiographic criteria after reduction: dorsal angulation >10°, radial inclination <15°, or radial shortening >3 mm). All fractures were amenable to treatment with all 3 surgical treatments. Patients with open fractures, bilateral fractures, prior DRF to the same wrist, or additional serious trauma were ineligible. We excluded patients with neurological conditions affecting upper extremity sensation or movement, comorbid conditions prohibiting surgery, serious neurological or psychiatric conditions precluding informed consent, and inability to complete study questionnaires and follow directions in English (or Chinese in Singapore).

Participants who opted for surgery were randomized to undergo VLPS, EFP, or CRPP. Randomization was stratified by site and was performed online.^18^ The treating surgeon had discretion regarding implant or fixator brand and the use of tourniquet, deep vein thrombosis prophylaxis, or prophylactic antibiotics. Recognizing that there will always be patients who prefer nonsurgical management, we created an observation group to include patients who met all the inclusion and exclusion criteria but did not want to undergo surgery. Observation group participants also provided written informed consent. The fractures of these participants were managed with casting. An initial short, arm fiberglass cast was replaced with a thermoplastic splint after a month. Follow-up care and hand therapy use for both surgical and casting participants were per institutional standard.

### Data Collection and Outcome Measures

Research team members not involved with patient care performed assessments at enrollment, 2 weeks, 6 weeks, 3 months, 6 months, 12 months, and 24 months after surgery or, for casting participants, after fracture. Participants completed the full Michigan Hand Outcomes Questionnaire (MHQ) at the 6-week through 24-month assessments. Only the pain domain of the MHQ was completed at enrollment and 2 weeks because all participants were immobilized in a cast or splint at this time point and, thus, were not performing any activities with the recently fractured wrist. The Medical Outcomes Study 36-Item Short-Form Health Survey (SF-36) was completed at all visits.^19^ At enrollment, participants provided demographic information and completed the Self-Administered Comorbidity Checklist.^20^ Participants were asked to report preinjury activity level using the Rapid Assessment of Physical Activity at enrollment and current activity at 24 months.^21,22^ Grip and lateral pinch strength and wrist arc of motion were measured at the 6-week through 24-month assessments. Deidentified digital copies of radiographs were sent to the Coordinating Center to measure radial height, radial inclination, volar or dorsal tilt, and ulnar deviation. Malunion was defined as dorsal or volar tilt greater than 10° from neutral, radial inclination less than 15°, or radial shortening greater than 3 mm.^15,23,24^

### Statistical Analysis

The Data Safety and Monitoring Board–approved statistical analysis plan is available in the study protocol (Supplement 1). The analytical cohort was intention-to-treat. The primary aim of WRIST was to compare MHQ summary and domain scores and clinical functional outcomes by treatment group 12 months after surgery or fracture.^17^ The present analysis represents the fourth aim of the funded proposal: to compare 24-month outcomes by the 4 treatment groups. We first reported crude means at 24 months for all outcomes by treatment group. We compared 24-month outcomes across the 4 groups using a mixed-effects model with follow-up data at all assessment times as the response variable.^25^ The model for each outcome variable included participants nested within sites as random intercepts, and treatment group indicators, time indicators, time by treatment group indicators, and baseline values of the outcome measure (or baseline MHQ pain scores for the analysis of MHQ summary and domain scores) were included as factors associated with outcomes. The model also included as covariates baseline factors found to be associated with missing 24-month follow-up data: age, race, smoking status, and baseline Rapid Assessment of Physical Activity score. Race was assessed because it has been associated with missing visits. Participants self-identified their race and other demographic information. Assuming missing-at-random, we expect the mixed model to provide unbiased estimates.^26^ Marginal means at month 24 based on the model were compared across the treatment groups. Because missing-at-random cannot be tested, we also assessed whether results across treatment groups depended on the pattern of missingness by graphically examining the means of outcome measures at each assessment time based on all observations (eFigure 1 in Supplement 2) and also by the missing data pattern (eFigure 2 in Supplement 2).

We had 2 secondary aims. First, we determined changes in patient-reported and functional outcomes between the 12- and 24-month assessments. For each of the outcome measures, means across groups at 12 and 24 months were compared using the aforementioned mixed model. Second, we evaluated whether outcomes differed between participants who experienced malunion vs those who did not. Malunion was defined on the basis of radiographic data at 24 months. For those without radiographic data at 24 months, we used the 12-month data because previous investigators have found that radiographic alignment does not change after 12 months.^15^ In WRIST, 92 participants had both 12- and 24-month radiographic data; malunion status did not change from 12 to 24 months in any of these 92 participants. We compared patient-reported outcomes (PROs) at 24 months between the 2 groups of those with malunion vs those without malunion using 2-sample *t* test. Significance was set at *P* < .05 for all analyses. We used Stata statistical software version 15.1 (StataCorp) for our analyses. Data analysis was performed from March 2019 to March 2021.

## Results

A total of 304 participants were enrolled in WRIST; 187 participants (62%) were randomized to undergo 1 of the 3 surgical procedures (VLPS, 65 participants; EFP, 64 participants; CRPP, 58 participants), and 117 participants (38%) opted for casting. Eight casting participants were found to be ineligible after enrollment and were excluded from the study. Of the 296 eligible participants, 256 (87%) were women, and their mean (SD) age was 71.1 (8.9) years. Assessments at 24 months were performed for 182 participants (160 women [87.9%]; mean [SD] age of 70.1 [8.5] years). Of the 114 participants who did not complete 24-month follow-up assessments, 28 withdrew consent (largely because of participant burden); 21 received diagnoses of terminal illness, entered a nursing home, or died; and 65 were lost to follow-up (Figure). Among persons who declined to be included in the study, 46% did not want to risk being randomized to EFP, and 4 enrolled participants who were randomized to EFP refused to have the procedure. Casting participants were more likely to miss 24-month assessments (65 participants [60%]) than randomized participants (49 participants [26%]), but there was no difference in the rate of missingness across the 3 randomized groups. Participants without 24-month follow-up assessments were also likely to be older (median [range] age, 68 [59-97] vs 73 [58-92] years), Asian (16 [14.0%] vs 8 [4.4%] participants), sedentary at baseline (21 [18.4%] vs 13 [7.1%] participants), and current smokers (18 [15.8%] vs 10 [5.5%] participants) (Table 1). Among those with 24-month follow-up assessments, casting group participants were older (eTable 1 in Supplement 2) and reported lower baseline pain (eTable 2 in Supplement 2) than randomized participants; we did not find any other baseline variables to be associated with treatment group.

**Figure.**
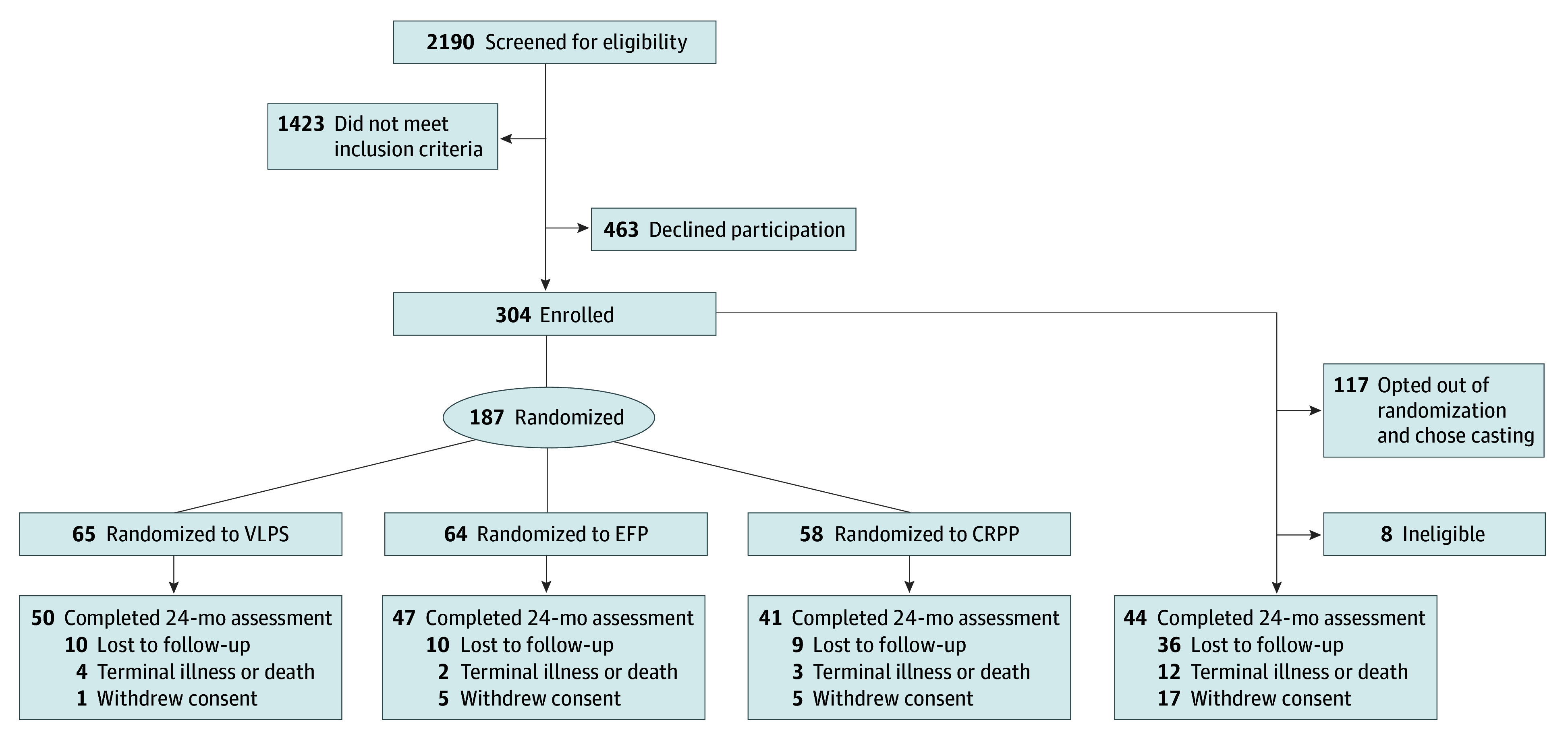
Participant Enrollment Flowchart CRPP indicates closed reduction with percutaneous pinning; EFP, external fixation with or without supplementary pinning; VLPS, volar locking plate system.

**Table 1.** Baseline Demographic and Clinical Characteristics by Completion of 24-Month Assessment

Characteristic	Participants, No. (%)		*P* value
	Completed 24-mo assessment (n = 182)	Did not complete 24-mo assessment (n = 114)	
Sex			
Female	160 (87.9)	96 (84.2)	.37
Male	22 (12.1)	18 (15.6)	
Age, y			
Mean (SD)	70.1 (8.5)	73.0 (9.3)	.02
Median (range)^a^	68 (59-97)	73 (58-92)	
Race			
Asian	8 (4.4)	16 (14.0)	.05^b^
Black	11 (6.0)	6 (5.3)	
White	159 (87.4)	90 (79.0)	
Other^c^	2 (1.1)	2 (1.8)	
Missing	2 (1.1)	0	
Education			
High school diploma, general equivalency diploma, or less	61 (42.1)	48 (42.1)	.13^b^
Vocational school, associate’s degree, or some college	52 (28.6)	35 (30.7)	
Bachelor’s degree or higher	64 (35.2)	29 (25.4)	
Missing	5 (2.8)	2 (1.8)	
Employment at baseline			
Full-time	30 (16.5)	16 (14.0)	.79^b^
Part-time	26 (14.3)	9 (7.9)	
Retired	115 (63.2)	66 (57.9)	
Receiving disability	5 (2.8)	2 (1.8)	
Unemployed	6 (3.3)	4 (3.5)	
Missing	0	17 (14.9)	
Annual household income, $			
<20 000	35 (19.2)	27 (23.7)	.03^b^
20 000-39 000	37 (20.3)	37 (32.5)	
40 000-59 999	33 (18.1)	17 (14.9)	
≥60 000	57 (31.3)	22 (19.3)	
Missing	20 (11.0)	11 (9.7)	
Functional status before injury			
Sedentary	13 (7.1)	21 (18.4)	.001^b^
Underactive	86 (47.3)	62 (54.4)	
Active	82 (45.1)	30 (26.3)	
Missing	1 (0.6)	1 (0.9)	
Smoking status			
Never	102 (56.0)	53 (46.5)	.01^b^
Former, <10 y	69 (37.9)	42 (36.8)	
Current	10 (5.5)	18 (15.8)	
Missing	1 (0.6)	1 (0.9)	
Comorbidities, No.			
Mean (SD)	3.4 (2.2)	3.7 (2.7)	.23
Median (range)	3 (0-11)	3 (0-12)	
Arbeitsgemeinschaft für Osteosynthesefragen class			
A1	2 (1.1)	0	.30^b^
A2	81 (44.5)	49 (43.0)	
A3	25 (13.7)	7 (6.1)	
C1	10 (5.5)	10 (8.8)	
C2	51 (28.0)	30 (26.3)	
C3	2 (1.1)	2 (1.8)	
Missing	11 (6.0)	16 (14.0)	
Ulnar styloid fracture			
Yes	83 (45.6)	42 (36.8)	.57^b^
No	88 (48.4)	55 (48.3)	
Missing	11 (6.0)	17 (14.9)	
Treatment			
Volar locking plate system	50 (27.5)	15 (13.2)	<.001
External fixation with or without supplementary pinning	47 (25.8)	17 (14.9)	
Closed reduction with percutaneous pinning	41 (22.5)	17 (14.9)	
Casting	44 (24.2)	65 (57.0)	

^a^ In the early phases of the study, 5 participants younger than 60 years were enrolled.

^b^ *P* values comparing those completing the 24-month assessment vs those who did not were calculated after excluding those with missing data.

^c^ Other includes American Indian/Alaska Native, Pacific Islander/Hawaii Native, multiple races, and all other races.

At 24 months, MHQ scores indicated a low level of disability; mean MHQ summary score of all participants was 85 (95% CI, 83-88), representing good overall hand function.^4^ Participants reported low pain (mean MHQ pain score, 13 [95% CI, 10-16]), good return of their activities of daily living (mean MHQ activities of daily living score, 88 [95% CI, 85-90]), and good satisfaction (mean MHQ satisfaction score, 82 [95% CI, 78-86]). We did not find any difference across 4 treatments in mean MHQ summary score (VLPS, 88 [95% CI, 83-92]; EFP, 83 [95% CI, 78-88]; CRPP, 85 [95% CI, 79-90]; and casting, 85 [95% CI, 79-90]; χ^2^_3_ = 1.44; *P* = .70) or in any MHQ domain scores, including pain score (χ^2^_3_ = 2.64; *P* = .45) at 24 months after adjusting for covariates (Table 2). For MHQ summary and domain scores, an 8-point difference or change is considered the minimal clinically important difference.^5,6^ We did not find any pairwise differences between any 2 treatment groups to be larger than 6 points in summary or pain domain scores. Our models included baseline factors associated with missing 24-month assessments. However, to further assess the association of potential biases from differential losses with follow-up at 24 months, eFigure 1 in Supplement 2 shows mean MHQ summary scores over time by treatment group using all available data, and eFigure 2 in Supplement 2 shows mean MHQ summary scores by the pattern of missingness for each treatment group. Longitudinal trends in MHQ summary scores do not appear to be associated with missing data pattern and do not show notable deviations across the 4 treatment groups.

**Table 2.** Descriptive Statistics for Primary and Secondary 24-Month Outcomes by Study Groups

Measures	VLPS (n = 50)	EFP (n = 47)	CRPP (n = 41)	Casting (n = 44)	*P* value^a^
Michigan Hand Outcomes Questionnaire, crude mean (95% CI)					
Summary	88 (83-92)	83 (78-88)	85 (79-90)	85 (79-90)	.70
Function	87 (82-92)	83 (77-89)	82 (77-88)	78 (72-85)	.34
Activities of daily living	91 (87-96)	85 (79-91)	89 (84-94)	85 (79-91)	.62
Work	87 (80-93)	85 (79-91)	88 (82-94)	86 (81-92)	.99
Pain	11 (5-17)	14 (8-19)	14 (8-20)	15 (9-22)	.45
Aesthetics	86 (80-93)	84 (77-90)	87 (80-93)	89 (83-94)	.97
Satisfaction	87 (80-94)	83 (76-90)	79 (70-89)	79 (70-88)	.20
Medical Outcomes Study 36-Item Short-Form Health Survey, crude mean (95% CI)					
Physical Component Score	46 (43-49)	46 (43-49)	49 (45-52)	46 (42-49)	.69
Mental Component Score	56 (53-58)	54 (51-57)	54 (51-57)	53 (50-56)	.29
Functional measures, crude mean (95% CI), % of uninjured hand or wrist					
Grip strength	90 (83-97)	81 (74-88)	89 (84-95)	85 (78-92)	.15
Pinch strength	99 (94-105)	92 (85-99)	99 (85-113)	102 (91-113)	.18
Flexion	91 (85-96)	94 (85-102)	89 (83-95)	83 (76-91)	.27
Extension	95 (88-101)	90 (85-95)	94 (90-99)	94 (87-101)	.54
Ulnar deviation	98 (91-105)	91 (83-100)	102 (90-115)	97 (87-107)	.66
Radial deviation	99 (87-112)	92 (83-100)	108 (92-124)	115 (96-133)	.05
Pronation	99 (98-100)	99 (97-101)	99 (97-101)	97 (90-104)	.84
Supination	104 (94-114)	95 (93-98)	94 (90-98)	94 (90-99)	.20
Malunion present, participants, No. (%)	4 (8.0)	8 (17.0)	4 (9.8)	26 (59.1)	<.001
Physical activity change from baseline, participants, No. (%)					
Increased	9 (18.0)	9 (19.2)	5 (12.2)	7 (15.9)	.70
Decreased	11 (22.0)	11 (23.4)	9 (22.0)	10 (22.7)	
No change	25 (50.0)	21 (44.7)	26 (63.4)	25 (56.8)	
Missing	5 (10.0)	6 (12.8)	1 (2.4)	2 (4.6)	

Abbreviations: CRPP, closed reduction with percutaneous pinning; EFP, external fixation with or without supplementary pinning; VLPS, volar locking plate system.

^a^ *P* values compare 24-month marginal means across treatment groups using a linear mixed-effects model with data at all follow-up time from 4 treatment groups. The models included categorical time indicators, treatment group indicators, interactions of treatment group by time indicators, and baseline covariates associated with missing 24-month assessment (age, baseline pain score, current smoking status, activity level [Rapid Assessment of Physical Activity], and race) as factors. For categorical variables, χ^2^ test was used.

PROs and clinical outcomes generally improved from 12 to 24 months across the 4 treatment groups. However, the increases based on MHQ summary score were neither statistically significant nor clinically relevant except for MHQ function and pain domain scores. Table 3 shows crude means at 12 and 24 months for all outcome measures in overall groups, and eTable 3 in Supplement 2 shows crude means by treatment groups. Averaged across groups, crude mean MHQ summary score increased from 82 (95% CI, 80-85) to 85 (95% CI, 83-88) (*P* = .12). Crude MHQ pain scores decreased from 19 (95% CI, 16-22) to 13 (95% CI, 10-16) (*P* = .001). Although improvement in pain did not differ significantly by treatment groups, estimated reductions were −7 points (*z* = −2.04; *P* = .04) in the VLPS group and −8 points (*z* = −2.26; *P* = .02) in the EFP group. Crude MHQ function scores improved from a mean of 77 (95% CI, 74-80) to 83 (95% CI, 80-86) (*P* < .001), with estimated improvement by 7 points both in the VLPS (*z* = 2.30; *P* = .02) and EFP (*z* = 2.01; *P* = .04) groups. The hand and wrist strength and motion of the participants reached nearly 100% of the contralateral uninjured hand and wrist by 24 months. Overall from 12 to 24 months, the estimated grip strength improvement was 8% (change in crude mean from 79% [95% CI, 77%-82%] to 86% [95% CI, 83%-90%]; *P* < .001), pinch strength improvement was 9% (change in crude mean from 89% [95% CI, 87%-91%] to 98% [95% CI, 93%-103%]; *P* < .001), and wrist flexion improvement was 7% (change in crude mean from 85% [95% CI, 82%-87%] to 90% [95% CI, 86%-93%]; *P* = .004). Differential improvement across treatment groups were found in grip strength, with the largest estimated improvement in the EFP group (10.3 points), and in pinch strength, with the largest estimated improvement in casting group (17.2 points) (eTable 3 in Supplement 2). There were no changes in quality of life as measured by the SF-36.

**Table 3.** Descriptive Statistics for Primary and Secondary Outcomes at 12-Month and 24-Month Follow-up, and Comparisons Between 12 to 24 Months Across the 4 Treatment Groups

Measures	12 Months	24 Months	*P* value^a^
Michigan Hand Outcomes Questionnaire score, crude mean (95% CI)			
Summary	82 (80-85)	85 (83-88)	.12
Function	77 (74-80)	83 (80-86)	<.001
Activities of daily living	85 (82-88)	88 (85-90)	.26
Work	82 (79-85)	86 (83-89)	.14
Pain	19 (16-22)	13 (10-16)	.001
Aesthetics	84 (82-87)	86 (83-89)	.59
Satisfaction	78 (75-82)	82 (78-86)	.06
Medical Outcomes Study 36-Item Short-Form Health Survey score, mean (95% CI)			
Physical Component Score	47 (45-48)	46 (45-48)	.51
Mental Component Score	55 (53-56)	54 (53-56)	.24
Functional measures, crude mean (95% CI), % of uninjured hand or wrist			
Grip strength	79 (77-82)	86 (83-90)	<.001
Pinch strength	89 (87-91)	98 (93-103)	<.001
Flexion	85 (82-87)	90 (86-93)	.004
Extension	92 (90-95)	93 (90-96)	.41
Ulnar deviation	89 (83-95)	97 (92-102)	.02
Radial deviation	100 (93-106)	103 (96-110)	.18
Pronation	98 (96-99)	99 (97-100)	.40
Supination	97 (94-99)	97 (94-100)	.82

^a^ *P* values were calculated by testing for the difference in means between month 12 and 24 averaged across the 4 treatment groups using a linear mixed-effects model with data at all follow-up time from all 4 treatment groups adjusting for categorical time indicators, treatment group indicators, and baseline covariates associated with missing 24-month assessment (age, baseline pain score, current smoking status, activity level [Rapid Assessment of Physical Activity], and race).

Malunion was experienced by 42 participants (23%) completing 24-month assessments. As expected, malunion varied significantly by treatment group; 26 participants (59.1%) in the casting group, 8 participants (17.0%) in the EFP group, 4 participants (9.8%) in the CRPP group, and 4 participants (8.0%) in the VLPS group experienced malunion (χ^2^_3_ = 43.6; *P* < .001) (Table 2). Comparisons of baseline characteristics showed participants with malunion to be significantly older than those without malunion (mean [SD] age, 74 [10.6] vs 69 [7.4] years; difference, 5 years; 95% CI, 2.1-7.8 years; *P* < .001) because casting participants had a higher rate of malunion (Table 4). We did not find other baseline characteristics to be associated with malunion. At 24 months, participants with malunion generally showed lower function than those without malunion, as measured by MHQ summary and domain scores, but no measures showed clinically or statistically meaningful difference (eTable 4 in Supplement 2). Although not significant, flexion and ulnar deviation were also notably worse in those with malunion. Finally, participants with malunion and those without did not vary in employment or physical activity at 24 months.

**Table 4.** Baseline Demographic and Clinical Characteristics by Malunion Status

Variables	Participants, No. (%)		*P* value^a^
	No malunion (n = 140)	Malunion (n = 42)	
Sex			
Female	122 (87.1)	38 (90.5)	.56
Male	18 (12.9)	4 (9.5)	
Age, y			
Mean (SD)	69 (7.4)	74 (10.6)	<.001
Median (range)	67 (59-92)	72 (59-97)	
Race			
Asian	6 (4.3)	2 (4.8)	.80
Black	7 (5.0)	4 (9.5)	
White	123 (88.6)	36 (85.7)	
Other^b^	2 (1.4)	0	
Missing	2 (1.4)	0	
Education			
High school diploma, general equivalency diploma, or less	46 (32.9)	15 (35.7)	.66
Vocational school, associate’s degree, or some college	40 (28.6)	12 (28.6)	
Bachelor’s degree or higher	49 (35.0)	15 (35.7)	
Missing	5 (3.6)	0	
Employment			
Full-time	25 (17.9)	5 (11.9)	.55
Part-time	22 (15.7)	4 (9.5)	
Retired	86 (61.4)	29 (69.1)	
Receiving disability	3 (2.1)	2 (4.8)	
Unemployed	4 (2.9)	2 (4.8)	
Annual household income, $			
<20 000	24 (17.1)	11 (26.2)	.61
20 000-39 000	27 (19.3)	10 (23.8)	
40 000-59 999	27 (19.3)	6 (14.3)	
≥60 000	46 (32.9)	11 (26.2)	
Missing	16 (11.4)	4 (9.5)	
Physical activity level before injury			
Sedentary	11 (7.9)	2 (4.8)	.65
Underactive	63 (45.0)	23 (54.8)	
Active	65 (46.4)	17 (40.5)	
Missing	1 (0.7)	0	
Smoking status			
Never	78 (55.7)	24 (57.1)	.95
Former	53 (37.9)	16 (38.1)	
Current	9 (6.4)	2 (4.8)	
Comorbidities, No.			
Mean (SD)	3.3 (2.2)	3.5 (2.2)	.60
Median (range)	3 (0-11)	3.5 (0-8)	
Arbeitsgemeinschaft für Osteosynthesefragen class			
A1	2 (1.4)	0	.89
A2	64 (45.7)	17 (40.5)	
A3	18 (12.9)	7 (16.7)	
C1	8 (5.7)	2 (4.8)	
C2	38 (27.1)	13 (31.0)	
C3	1 (0.7)	1 (2.4)	
Missing	9 (6.4)	2 (4.8)	
Ulnar styloid fracture			
Yes	67 (47.9)	16 (38.1)	.54
No	65 (46.4)	23 (54.8)	
Missing	8 (5.7)	3 (7.1)	

^a^ *P* values are from testing for the difference between those with vs without malunion using *t* test for continuous variables and χ^2^ test for categorical variables.

^b^ Other includes American Indian/Alaska Native, Pacific Islander/Hawaii Native, multiple races, and all other races.

## Discussion

In this study, participants aged 60 years and older experienced little disability, low pain, good self-reported and measured function, and high quality of life 24 months after DRF. We reject our hypothesis because our study showed that 24-month outcomes did not differ by treatment. Earlier analyses of WRIST data have shown similar results; early in the follow-up period, participants treated with VLPS reported better ability to perform activities of daily living and satisfaction and recovered more strength and wrist motion, but by 6 months any differences had disappeared and all participants showed satisfactory outcomes.^17^ Similar results have been demonstrated by other investigators.^8,27,28^ We found all hand outcomes, including the MHQ summary measure, to have generally improved from 12 to 24 months across treatment groups; however, only function and pain improved significantly between 12- and 24-month assessments, and the magnitude of the improvement was not clinically meaningful. In addition, we did not find improvements seen in PROs to differ by treatment groups.

To our knowledge, there are only 8 published reports of 24-month or longer outcomes in older populations, and, of those, only 2 reported intermediate time points.^8,9,10,11,12,13,14,16^ Furthermore, no study conducted a concurrent comparison of all 4 currently accepted treatments. Arora et al^8^ found no changes in radiographic alignment between 12-week and 4-year assessments of patients aged 70 years and older treated with casting or open reduction and internal fixation. Aktekin et al^11^ showed that wrist extension and ulnar deviation were significantly better in patients treated with EFP compared with casted patients who were aged 65 years or older. Sirniö et al^9^ reported no changes in Disabilities of the Arm, Shoulder, and Hand score between 12 and 24 months in patients aged 50 years and older who were treated with VLPS. Our results are similar to studies^29,30,31,32^ of younger adults, in which no change in PROs including pain was observed after 12 months. WRIST participants also did not show significant improvement between 12 and 24 months in most PROs, but exhibited improvements in hand function and pain. Both flexion and grip strength improved, although neither reached the level of the contralateral, uninjured wrist or hand. This corroborates with previous studies that reported slower recovery after DRF for older patients.^33,34^

Barton et al^12^ reported an association between radial shortening and Patient-Rated Wrist Evaluation score at a mean of 29 months after treatment with k-wire fixation among patients aged 55 years and older. Likewise, Brogren et al^35^ reported worse Disabilities of the Arm, Shoulder, and Hand scores among adult patients with malunion diagnosed 2 years after treatment with casting or pinning. Our results are more similar to previous investigations^10,12,13^ that reported no association between fracture alignment and PROs in older patients. The WRIST trial provided evidence that, although more than 50% of casting participants experience malunion, casting provides long-term outcomes that are indistinguishable from those of participants treated with surgical procedures. Furthermore, casting patients experienced these outcomes with fractures of equal severity compared with surgical participants.

Although the 24-month EFP outcomes were no different than those for the other treatments, for Colles type fractures that are common in older adults with osteoporosis, EFP is not ideal. EFP is inadequate in restoring anatomic alignment and is cumbersome for older adults. Furthermore, pin site infection is a constant concern with the exposed pins. Finally, the WRIST investigators experienced difficulty enrolling eligible participants because they did not want to risk randomization into EFP. Avoiding randomization was cited by 46% of declining patients, and 4 enrolled participants who were randomized to EFP refused to have the procedure. However, EFP is still applicable for severely comminuted fractures or for those requiring temporary stabilization during infection.

### Limitations

One limitation of this study is low participant retention, in particular for the casting group. Participants who did not complete follow-up assessments were also older, less active, current smokers, more likely to be Asian, and tended to be in worse health, and their exclusion may result in an overestimation of any improvements between 12 and 24 months. Despite adjusting for baseline characteristics that are associated with missing 24-month outcomes, the differential missingness associated with these measured and unmeasured baseline characteristics could have made our results appear more favorable for casting than may actually have been the case. Conversely, patients who did not return for 24-month assessments may have done so because they were satisfied with their recovery, thus underestimating 12- to 24-month improvements. Despite the limitation, similar profiles in mean MHQ summary scores over time across missing data patterns within each treatment groups suggest no evidence for biases.

## Conclusions

In this randomized clinical trial, there were no differences among the 4 treatment groups according to outcomes 24 months after fracture. Therefore, long-term outcomes are not a factor in selecting the optimal DRF treatment. The insight from these 24-month data is that older patients who chose nonoperative treatment adapted to their deformity and functioned similarly to those who chose an operative treatment, despite malunion. This effect was maintained at 2 years, which assures the lack of deterioration of overall function over time. If older patients understand the ramification of malunion and observed wrist deformity, it is appropriate to offer patients casting as a rational treatment option over the need for ideal radiographic correction with an operative approach.

## Notes

Supplement 1.
Trial Protocol

Supplement 2. **eTable 1.** Baseline Demographic and Clinical Characteristics by Treatment Groups in Those Who Completed 24-Months Assessment **eTable 2.** Descriptive Statistics for Baseline Values of Outcomes by Treatment Groups **eTable 3.** Unadjusted Means (95% Confidence Intervals) for Primary and Secondary Outcomes at 12- and 24-Month by Treatment Groups **eTable 4.** Descriptive Statistics for Primary and Secondary Outcomes at 24 Months by Malunion Status **eFigure 1.** MHQ Summary Scores Over the 24 Months Follow-up Assessment Times by Treatment Groups Using Data From All Cases **eFigure 2.** MHQ Summary Scores by the Pattern of Missingness, Separately for Each Treatment Group

Supplement 3.
WRIST Group Members

Supplement 4.
Data Sharing Statement
