# Supplementary material for: Comparison of 24-Month Outcomes After Treatment for Distal Radius Fracture: The WRIST Randomized Clinical Trial
Source: JAMA Netw Open. 2021 Jun 17;4(6):e2112710. doi: 10.1001/jamanetworkopen.2021.12710 (PMC12507456; doi:10.1001/jamanetworkopen.2021.12710)
Supplement: Supplement 1. — Trial Protocol [file jamanetwopen-e2112710-s001.pdf]

A Clinical Trial for the Surgical Treatment of Distal Radius Fracture in the Elderly: Wrist and Radius Injury Surgical Trial (WRIST)

Protocol Number: R01AR062066-01A1

National Clinical Trial (NCT) Identified Number: NCT01589692

Principal Investigator: Kevin C Chung, MD, MS

IND/IDE Sponsor: not applicable

Funded by: National Institute of Arthritis and Musculoskeletal and Skin Diseases and National Institute on Aging

Version Number: v7, 03April2018

Summary of Changes from Previous Version:

**Affected Section(s)**	**Summary of Revisions Made**	**Rationale**
All	Correcting formatting prior to manuscript submission	Protocol submission is required and over time revisions have altered formatting

Table of Contents

STATEMENT OF COMPLIANCE 1
1 PROTOCOL SUMMARY 2
1.1 Synopsis 2
1.2 Schema 3
1.3 Schedule of Activities (SoA) 4
2 INTRODUCTION 5
2.1 Study Rationale 5
2.2 Background 6
2.3 Risk/Benefit Assessment 12
2.3.1 Known Potential Risks 12
2.3.2 Known Potential Benefits 13
2.3.3 Assessment of Potential Risks and Benefits 13
3 OBJECTIVES AND ENDPOINTS 14
4 STUDY DESIGN 15
4.1 Overall Design 15
4.2 Scientific Rationale for Study Design 16
4.3 Justification for Dose 16
4.4 End of Study Definition 16
5 STUDY POPULATION 17
5.1 Inclusion Criteria 17
5.2 Exclusion Criteria 17
5.3 Lifestyle Considerations 17
5.4 Screen Failures 17
5.5 Strategies for Recruitment and Retention 18
6 STUDY INTERVENTION 19
6.1 Study Intervention(s) Administration 19
6.1.1 Study Intervention Description 19
6.1.2 Dosing and Administration 19
6.2 Preparation/Handling/Storage/Accountability 19
6.2.1 Acquisition and accountability 19
6.2.2 Formulation, Appearance, Packaging, and Labeling 19
6.2.3 Product Storage and Stability 19
6.2.4 Preparation 19
6.3 Measures to Minimize Bias: Randomization and Blinding 19
6.4 Study Intervention Compliance 20
6.5 Concomitant Therapy 21
6.5.1 Rescue Medicine 21
7 STUDY INTERVENTION DISCONTINUATION AND PARTICIPANT DISCONTINUATION/WITHDRAWAL 21
7.1 Discontinuation of Study Intervention 21
7.2 Participant Discontinuation/Withdrawal from the Study 21
7.3 Lost to Follow-Up 21
8 STUDY ASSESSMENTS AND PROCEDURES 22
8.1 Efficacy Assessments 22
8.2 Safety and Other Assessments 26
8.3 Adverse Events and Serious Adverse Events 26

8.3.1 Definition of Adverse Events (AE) 26
8.3.2 Definition of Serious Adverse Events (SAE) 26
8.3.3 Classification of an Adverse Event 26
8.3.4 Time Period and Frequency for Event Assessment and Follow-Up 27
8.3.5 Adverse Event Reporting 27
8.3.6 Serious Adverse Event Reporting 27
8.3.7 Reporting Events to Participants 27
8.3.8 Events of Special Interest 27
8.3.9 Reporting of Pregnancy 28
8.4 Unanticipated Problems .... **Error! Bookmark not defined.**
8.4.1 Definition of Unanticipated Problems (UP) .... **Error! Bookmark not defined.**
8.4.2 Unanticipated Problem Reporting .... **Error! Bookmark not defined.**
8.4.3 Reporting Unanticipated Problems to Participants .... **Error! Bookmark not defined.**
9 STATISTICAL CONSIDERATIONS 28
9.1 Statistical Hypotheses 28
9.2 Sample Size Determination 29
9.3 Populations for Analyses 30
9.4 Statistical Analyses 31
9.4.1 General Approach 31
9.4.2 Analysis of the Primary Efficacy Endpoint(s) 31
9.4.3 Analysis of the Secondary Endpoint(s) 33
9.4.4 Safety Analyses 35
9.4.5 Baseline Descriptive Statistics 35
9.4.6 Planned Interim Analyses 35
9.4.7 Sub-Group Analyses 35
9.4.8 Tabulation of Individual participant Data 36
9.4.9 Exploratory Analyses 36
10 SUPPORTING DOCUMENTATION AND OPERATIONAL CONSIDERATIONS 37
10.1 Regulatory, Ethical, and Study Oversight Considerations 37
10.1.1 Informed Consent Process 37
10.1.2 Study Discontinuation and Closure 37
10.1.3 Confidentiality and Privacy 38
10.1.4 Future Use of Stored Specimens and Data 38
10.1.5 Key Roles and Study Governance 39
10.1.6 Safety Oversight 39
10.1.7 Clinical Monitoring .... **Error! Bookmark not defined.**
10.1.8 Quality Assurance and Quality Control .... **Error! Bookmark not defined.**
10.1.9 Data Handling and Record Keeping 40
10.1.10 Protocol Deviations 42
10.1.11 Publication and Data Sharing Policy 43
10.1.12 Conflict of Interest Policy .... **Error! Bookmark not defined.**
10.2 Additional Considerations .... **Error! Bookmark not defined.**
10.3 Abbreviations 43
10.4 Protocol Amendment History 45
11 REFERENCES 46

STATEMENT OF COMPLIANCE

(1) The trial will be carried out in accordance with International Conference on Harmonisation Good Clinical Practice (ICH GCP) and the following:

- United States (US) Code of Federal Regulations (CFR) applicable to clinical studies (45 CFR Part 46, 21 CFR Part 50, 21 CFR Part 56, 21 CFR Part 312, and/or 21 CFR Part 812)

National Institutes of Health (NIH)-funded investigators and clinical trial site staff who are responsible for the conduct, management, or oversight of NIH-funded clinical trials have completed Human Subjects Protection and ICH GCP Training.

The protocol, informed consent form(s), recruitment materials, and all participant materials will be submitted to the Institutional Review Board (IRB) for review and approval. Approval of both the protocol and the consent form must be obtained before any participant is enrolled. Any amendment to the protocol will require review and approval by the IRB before the changes are implemented to the study. In addition, all changes to the consent form will be IRB-approved; a determination will be made regarding whether a new consent needs to be obtained from participants who provided consent, using a previously approved consent form.

1 PROTOCOL SUMMARY

1.1 SYNOPSIS

**Title:**	A Clinical Trial for the Surgical Treatment of Distal Radius Fracture in the Elderly: Wrist and Radius Injury Surgical Trial (WRIST)
**Study Description:**	The goal of this 24-center randomized controlled trial is to compare the physical, functional and quality of life outcomes of three different surgical methods for treating distal radius fractures in the elderly: close reduction and percutaneous pinning (CRPP), external fixation with or without percutaneous pinning (EFP) and internal fixation with volar locking plates. We will also follow a cohort of patients who opt for the non-surgical treatment, close reduction and casting, to examine and compare the same outcomes following non-surgical treatment.
**Objectives:**	Primary Objective: To compare outcomes of DRFs in patients age 60 years and older treated with VLPS with those treated with EFP, CRPP, and casting<br>Secondary Objectives: to compare the trend of recovery for DRFs treated with VLPS with the other fixation techniques and to determine predictors of outcomes
**Endpoints:**	Primary Endpoint: Michigan Hand Outcomes Questionnaire Summary score at 12-month assessment<br>Secondary Endpoints: Michigan Hand Outcomes Questionnaire Subdomain scores, SF-36, RAPA, hand strength, wrist range of motion
**Study Population:**	Community-dwelling adults age 60 years or older presenting to participating sites' emergency or urgent care department with isolated closed distal radius fracture
**Phase:**	not applicable
**Description of Sites/Facilities Enrolling Participants:**	24 hospitals or health systems in the US, Canada, and Singapore
**Description of Study Intervention:**	Participants will be randomized to receive open reduction and internal fixation with a volar locking plate, closed reduction and external fixation with a bridging external fixator device with or without supplemental k-wire fixation, or closed reduction and percutaneous pinning with k-wires. Participants in the observation arm with be treated with closed reduction and casting.
**Study Duration:**	92 months. April 2013-December 2019
**Participant Duration:**	24 months

138 ## 1.2 SCHEMA

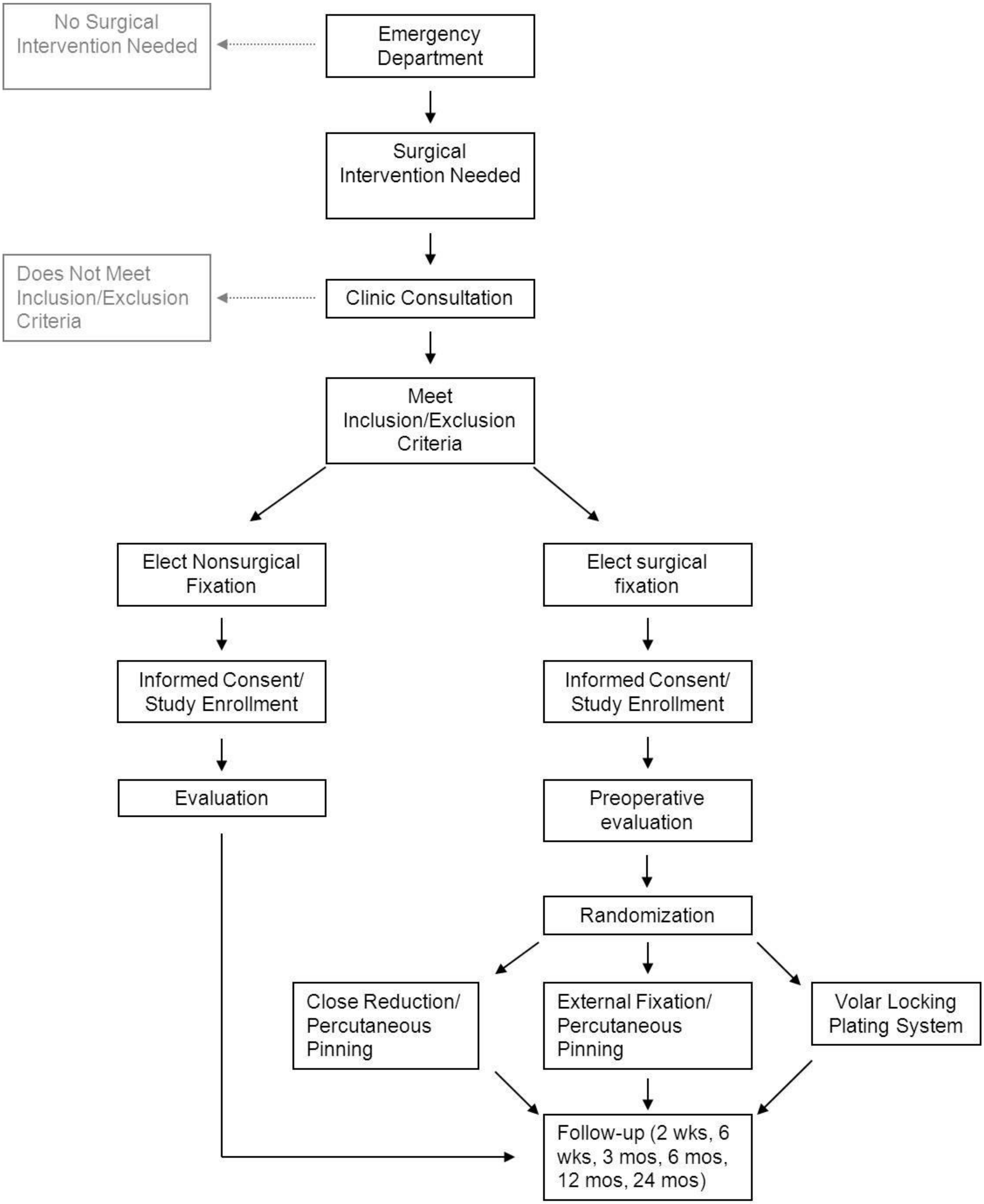

1.3 SCHEDULE OF ACTIVITIES (SOA)

**Visit number**	**1**	**2**	**3**	**4**	**5**	**6**	**7**	**8**	**9**
**Time (following surgery / closed reduction)**	**Screening**	**Enrollment**	**Surgery**	**2 wks (+1 week)**	**6 wks (+/- 1week)**	**3 mos (+/- 2 weeks)**	**6 mos (+/- 2 weeks)**	**12 mos (+/- 8 weeks)**	**24 mos (+/- 8 weeks)**
**Study Activities**									
**X-ray**		✓		✓	✓	✓	✓	✓	✓
**Inclusion/Exclusion**	✓								
**MMSE**		✓							
**Informed Consent Document**	✓								
**Randomization**	✓								
**Participant Demographics**		✓							
**Comorbidity Checklist**		✓							
**RAPA**		✓							✓
**SF-36**		✓		✓	✓	✓	✓	✓	✓
**Pain Domain of MHQ**		✓		✓					
**Surgery Data Form**			✓						
**General Health Questionnaire**				✓	✓	✓	✓	✓	✓
**Fingertip to Palmar Crease Distance Form**				✓	✓				
**Complication Checklist**				✓	✓	✓	✓	✓	✓
**Hand Therapy Data Form**				✓	✓	✓	✓	✓	✓
**MHQ (All Domains)**					✓	✓	✓	✓	✓
**Hand Function Data Form**					✓	✓	✓	✓	✓
**Participant Payment**		✓		✓	✓	✓	✓	✓	✓
**Adverse Event Collection** (if necessary)		✓	✓	✓	✓	✓	✓	✓	✓
**Protocol Deviation Collection** (if necessary)		✓	✓	✓	✓	✓	✓	✓	✓
	RAPA = Rapid Assessment of Physical Activity; MHQ = Michigan Hand Outcomes Questionnaire; SF-36 = Short-Form 36 Questionnaire; MMSE = Mini Mental State Examination								

2 INTRODUCTION

2.1 STUDY RATIONALE

Distal radius fractures (DRFs) are a public health concern. They represent the second most common fracture suffered by the elderly, after hip fractures. As the population of the United States ages, the number of persons sustaining this injury will increase as well. In the US, approximately 10% of 65-year-old white women will suffer a DRF during their remaining lifetime, most commonly due to osteoporosis.[1] The annual direct health care expenditure on osteoporotic forearm fractures in the US is at least $385 million.[2,3]

The optimal treatment strategy for DRFs in the elderly population remains controversial and undefined. Currently, there are four main types of treatment: closed reduction and casting, percutaneous pin fixation, external fixation ± pin fixation, and internal fixation. The less invasive techniques, which include closed reduction and casting, percutaneous pin fixation and external fixation ± pin fixation do not require extensive dissection, but may not adequately restore anatomic alignment. This is because osteoporotic bone in the elderly may be prone to collapse and displacement when these less rigid fixation methods are used.[4,5] Furthermore, conservative techniques may have recovery periods as long as 6 to 9 months. With internal fixation, the elderly may require less immobilization time and be able return to normal activities sooner. Recent advances in internal fixation technique, namely volar plates with locking screws, have made this treatment increasingly popular.[5-7] The distinct advantage of the volar locking plating system (VLPS) is its inherent stability, which allows most patients to begin wrist motion immediately after surgery, without concern of hardware failure or fracture collapse. However, the internal fixation techniques require more surgical dissection and may be technically challenging. The invasiveness of the operation also may be associated with more complications. Despite these uncertainties in treating DRFs in the elderly, relatively few studies have been conducted to examine this issue. Currently available studies, mainly small trials with inadequate power, do not compare the less invasive techniques with VLPS or do not exclusively evaluate fractures in the elderly.[8-17] The main aim of this proposal is to conduct a randomized controlled clinical trial comparing VLPS to closed reduction/percutaneous pinning (CRP) and external fixation± pin fixation (EFP) in treating unstable DRFs in the elderly.

2.2 BACKGROUND

Epidemiology

DRFs are the most common fractures of the human skeleton, accounting for 15-20% of all fractures seen by physicians.[18-21] The incidence of DRFs has a bimodal distribution across age groups, with peaks in the 2nd and 3rd decades of life and increasing steadily after the 5th decade due to osteoporosis. (Figure 1).[18,19]

Figure 1. Incidence of Distal Radius Fractures per 10,000 Persons

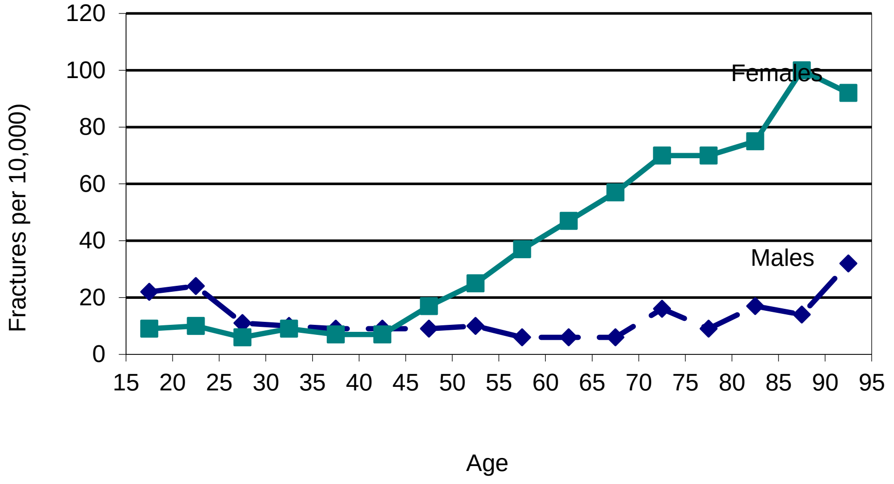

DRF is an injury that has higher incidence in females with osteoporotic bone (odds ratio of 7.0 when compared with males). Unlike DRFs in the younger population that are mainly a result of high energy trauma, DRFs in the elderly most often occur because of falls from standing height onto an outstretched hand. The weak metaphyseal bone of osteoporotic patients is insufficient to support the hand during a fall. In contrast to a hip fracture that occurs in an infirmed and less agile patient who is unable to use his/her hand to break the fall, DRF is more common in a functional patient who is able to break the fall with the hand.[22]

Public Health Importance and Economic Burden

There are over 37 million individuals 65 years of age and older in the US, an increase of nearly 2 million over the past five years.[23] This number will continue to rise as Baby Boomers age. Given that the incidence of DRF in the US population over the age of 65 is reported as 80 to 100 per 10,000 [1,24], we extrapolate that nearly 372,000 individuals 65 years of age and over sustain this type of fracture every year.

The economic and social impact of this injury is substantial. The annual cost of treating DRFs in the elderly has been estimated from $385 million to $535 million.[2,3] Added to these costs are the direct expenses of medical treatment, which include physician fees, facility fees, surgical equipment, and the cumulative costs of a structured rehabilitation program supervised by hand therapists. The non-monetary costs are great as well. While pain and disability related to DRFs is generally short-lived in younger patients, the impairment in the elderly can be long-lasting and greatly affect independent living.[25] Elderly patients do not adapt to immobilization as well as younger patients. The prolonged

immobility renders the elderly to be dependent on others to complete everyday tasks and hampers their participation in social activities.[26] Disability can last long after the immobilization period, as much as 18 months or longer after injury.[27] Finally, the feeling that one is a burden to others can have a great emotional impact on elderly individuals.[25,28]

Preliminary Studies

The PI and his team at the University of Michigan strive to provide evidence-based data to guide the treatment of distal radius fracture. For the past three years, we have collected a prospective cohort of patients who were treated using the VLPS technique. Patients with unstable DRFs were enrolled consecutively. This study generated four publications to (1) define outcomes of VLPS, (2) establish the responsiveness of Michigan Hand Outcomes Questionnaire (MHQ) for DRF, (3) identify predictors of functional outcomes and (4) compare outcomes in the elderly to with a younger cohort.

In addition to this preliminary work, the PI has finished conducting an NIH-sponsored multicenter prospective study on rheumatoid hand arthroplasty and has written a guide on how to conduct clinical trials.[29] The PI and his collaborators are leading experts in clinical research in hand surgery and will form a team of methodology experts during the course of this planning period to assure a successful R01 proposal. The PI has secured participation in this project from the Michigan Institute for Clinical and Health Research (MICHR), which the PI serves as a faculty member and the Center for Statistical Consultation and Research (CSCAR), which Dr. Kim, our statistician, is a faculty member. Both organizations are structured by the University of Michigan to assist in clinical trials, and their involvement in this trial will assure the highest level of expertise for the successful conduct of this project.

Responsiveness of the MHQ for DRFs

Within the prospective cohort study, we performed a nested outcomes study to determine the responsiveness of the MHQ for DRFs. This study demonstrated that the MHQ is responsive for DRF.[30] The MHQ, which was designed by the PI, is a hand-specific outcomes questionnaire that contains 57-items that cover six domains: (1) overall hand function, (2) Activities of Daily Living (ADLs), (3) pain, (4) work performance, (5) aesthetics, and (6) patient satisfaction.[31,32]

The validity and responsiveness of the MHQ has been proven for a variety of common hand conditions.[33-38] For example, the responsiveness of the MHQ was tested pre- and post-operatively in patients having carpal tunnel release and was found to have greater responsiveness than the Disabilities of the Arm, Shoulder and Hand (DASH) questionnaire (a general upper extremity outcomes tool) and similar responsiveness to the Carpal Tunnel Questionnaire.[39]

The MHQ was administered to 96 DRF patients and physical measures were assessed at 3, 6 and 12 months after surgery. We found all six domains of the MHQ to have a standard response mean >0.8, indicating a highly responsive questionnaire for DRFs. The MHQ is much more responsive than physical measures, including grip strength and arc of motion. However, grip strength was found to be a

responsive measure in the physical testing for this injury.[30] Because of the responsiveness of the MHQ, we will use the MHQ as the principal outcomes tool in the proposed clinical trial.

Outcomes of DRFs using VLPS

In a subsequent study, we established VLPS to be an effective treatment for unstable DRFs. We treated 161 patients with VLPS over the course of two years. Patients' outcomes were measured at 3, 6 and 12 months after the surgical procedure. Outcome measures were the same as in previous studies (grip strength, lateral pinch strength, active wrist arc of motion, the Jebsen-Taylor test, radiographic parameters and the MHQ).[40]

Three months after the surgical procedure, we found that patients attained over 80% function, when compared to the opposite, uninjured wrist. The rapid recovery of function demonstrated that VLPS is a promising surgical technique and supported the wide enthusiasm for the use of this procedure for treatment of unstable DRFs. Given that VLPS is effective in the general population, its effectiveness in the elderly must be demonstrated when compared to the traditional techniques (EFP and CRP).

Predictors of Functional Outcomes in Patients treated with VLPS

Determining predictors of functional outcomes for patients with DRFs is difficult because prior studies have had small sample sizes, inconsistency in the follow-up period, retrospective study design and heterogeneous treatment options. This prospective study defined the importance of both patient demographic factors and adequacy of fracture reduction in influencing outcomes for a cohort of 79 patients undergoing VLPS treatment. We found that increased age and lower income were associated with poorer long-term outcomes. In addition, fracture displacement was associated with poor short-term outcomes.[41]

Defining predictors of functional outcomes is important to help surgeons identify patients at risk for poor outcomes so that interventional strategies can be initiated early in the recovery. Experience gained in this study is applicable in the planning of the proposed multicenter clinical trial. In this trial, we will perform subgroup analysis in an effort to define predictors of outcomes in the elderly in each of the treatment arms.

Outcome Study of DRFs in the Elderly

Because the VLPS technique is a potentially promising treatment in the elderly, we performed a case-control study to compare outcomes of those over 65 years to those 20-40 years of age treated using this technique. Prior retrospective studies have demonstrated the effectiveness of the VLPS technique in treating elderly fractures.[5,6] In our prospective study, patients were evaluated at 3, 6 and 12 months after surgery with standard physical testing and the MHQ. This comparison indicated no statistically significant difference between elderly patients and young patients in grip strength, active wrist arc of motion or the MHQ at the three testing intervals (Table 1).[42] The rate of recovery, as shown in Figure 5, demonstrated no statistically significant difference between the two groups.[42] This study confirmed that

this new technique provides adequate outcomes in the elderly that are comparable to the younger sample.

**Table 1. Outcome Measures by Age Group (Mean ± Standard Deviation)**						
	**3-month Post-Operative**		**6-month Post-Operative**		**12-month Post-Operative**	
	**Young**	**Elderly**	**Young**	**Elderly**	**Young**	**Elderly**
**Active Wrist Arc of Motion**	**86% ± 12%**	**81% ± 10%**	**89% ± 12%**	**84% ± 17%**	**92% ± 11%**	**95% ± 17%**
**Grip Strength**	**66% ± 19%**	**44% ± 28%**	**78% ± 22%**	**67% ± 35%**	**80% ± 22%**	**77% ± 31%**
**MHQ**	**77 ± 16**	**77 ± 15**	**83 ± 17**	**81 ± 17**	**82 ± 18**	**85 ± 18**
The MHQ is based on a scale of 0 to 100. The higher the scores, the better the subject's hand performance. Arc of Motion and Grip Strength are measured as a percentage of uninjured opposite hand.						

Figure 5. Mean MHQ Score by Age Group

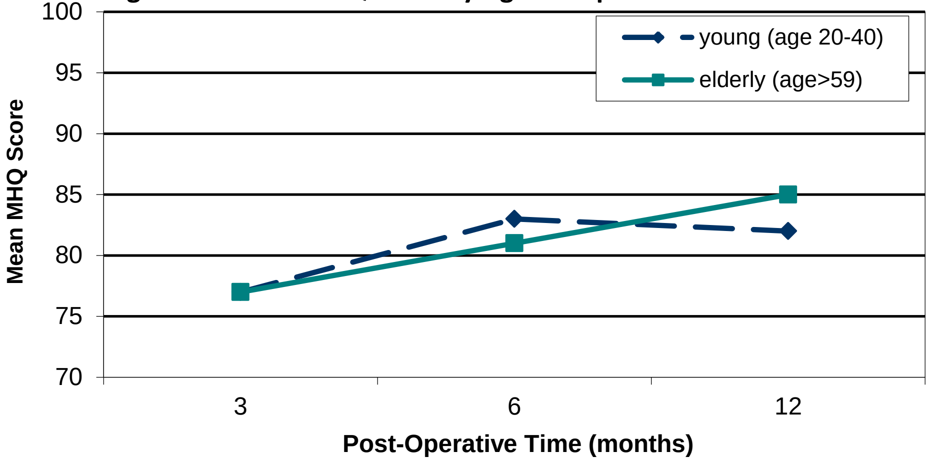

We will extend our experience from these prior studies to perform a multicenter clinical trial to compare this new intervention to established techniques (EFP and CRP) in the elderly. Although the VLPS technique is a promising treatment, its outcomes, complication rate and cost have not been studied in a larger cohort of subjects in a clinical trial design. This study will be the first of its kind to define the most optimal treatment for elderly DRFs.

Economic Analysis of DRF treatment

For Aim 5, we will perform an economic analysis comparing the three treatment arms. Economic analysis has become an important tool for determining which treatment strategy will provide the best outcome for the cost. In the PI's systematic review of over 3,000 Hand Surgery outcomes studies during the past 17 years, he found less than 1% of these publications were economic analysis studies.[43] When considering that the main impetus of the outcomes movement is to improve the quality of health care at

a reasonable cost, the PI concluded that the Hand Surgery specialty must conduct more economic analysis projects in the future.[43]

The PI has conducted cost-utility analyses on the treatment of two common conditions affecting the hand: carpal tunnel syndrome[44] and scaphoid fracture.[45] Derivatives of cost and quality-adjusted life-year (QALY) data will be important for the national health policy agenda because policy-makers evaluate the feasibility of funding support based on these two factors.

Epidemiology of Distal Radius Fracture in the US Elderly

The PI has recently begun a study to examine the current treatment of DRFs in the US elderly population by examining the Medicare data. This data support the need for a clinical trial examining the outcomes of VLPS in the elderly. Although closed reduction is still the dominant fixation method for the elderly, internal fixation has been steady growing in popularity, with the largest increases coming in recent years. This growth is despite any formal trial demonstrating internal fixation's effectiveness in the elderly. The data indicate that this study is timely in evaluating a new technology against the traditional approaches.

Figure 6. Rate of Use, by Fixation Method

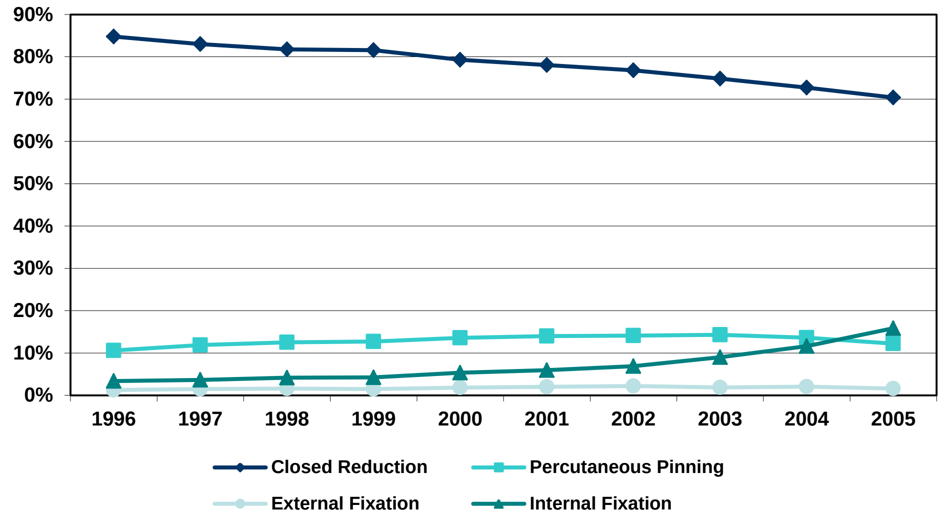

Study Impact

DRF is ubiquitous in our society, and mismanagement of this injury will lead to long-term functional problems. Evidence-based medicine has moved to the forefront as patients, physicians, funding agencies, and health-care policymakers are demanding high-quality research to enhance their understanding of healthcare decisions. Proponents of evidence-based medicine want physicians to either be armed with research information or to be able to successfully search for the evidence. They want physicians to "be able to understand the patient's circumstances or predicament; to identify knowledge gaps, and frame questions to fill those gaps; to conduct an efficient literature search; to critically appraise the research evidence; and to apply that evidence to patient care."[46] In spite of these

demands, appropriate clinical studies—from which evidence-based practice standards can be derived—are often in short supply. This is particularly true in surgical subspecialties. This was exemplified in a review conducted by the Journal of Hand Surgery, the premier journal of the specialty, which examined published studies in the field. This review noted that of 3,107 articles, abstracts, and letters reviewed from 111 issues of the Journal, there were only 25 controlled clinical trials and 8 randomized controlled trials.[47] Many of the studies published in hand surgery journals were markedly underpowered because of a lack of formal sample size calculation prior to conducting projects.[48,49] In addition, the PI has shown that 92% of the outcomes papers in the Journal of Hand Surgery have level 1 impact based on the Agency for Healthcare Research and Quality criteria.[43] A level 1 impact study confirmed the effectiveness of existing treatment without changing physician or patient practices to enhance the overall quality of care.[50] In a commentary to the readership of the Journal of Hand Surgery, the PI stressed the importance of enhancing the rigor of clinical research in hand surgery.[51] To develop evidence-based approaches to the treatment of these conditions, clinical scientists in the hand surgery field must first produce high-quality clinical studies.

There is a golden period around the introduction of a new intervention to fully evaluate its effectiveness. Once physicians have established a practice pattern, it is difficult for them to change and accept alternative treatments. Because of increasing acceptance of VLPS by hand surgeons, the appropriate time to perform a clinical trial comparing this new technology to existing technology is now.

2.3 RISK/BENEFIT ASSESSMENT

2.3.1 KNOWN POTENTIAL RISKS

All randomized patients may encounter the risks of anesthesia, which are RARE. These include: damage to teeth, mouth, throat or vocal cords, nerve or eye damage, drug reaction, slowing or stopping of breathing, failure of the anesthetic or sedation analgesia, cardiac arrest, risks that cannot be predicted, permanent disability or even death.

All randomized patients may encounter the general risks of distal radius fracture repair surgery, which are INFREQUENT. These include infection, pain, bleeding, persistent scar, deformity, nerve injury, tendon injury, decreased range of motion, malunion or nonunion, future arthritis (if fracture involves joint) and the need for further surgery.

All randomized patients may encounter the risk that the technique that the subject is randomized to will not be the appropriate one for them and the results inferior to those chosen outside the research context. This risk is RARE. All three techniques are considered standard procedures in the treatment of DRFs. All the surgeons participating in the study are experts in the treatment of DRFs and are equally comfortable performing each technique.

Procedure-specific risks

<u>Percutaneous Pinning</u>
Pin site infection requiring antibiotics: INFREQUENT
Pin migration: INFREQUENT
Tendon rupture: RARE

<u>External fixation</u>
Infection requiring antibiotics: INFREQUENT
Persistent wrist stiffness: INFREQUENT
Sensory nerve lesion: RARE
Tendon rupture: RARE

<u>Internal Fixation</u>
Wound infection requiring antibiotics: INFREQUENT
Painful hardware requiring removal: RARE

We will try to avoid all risks associated with surgery by employing best practices regarding pre-operative risk screening, infection control, and all other aspects of the surgical process.

All patients may encounter the risk of loss of privacy or confidentiality. This risk is RARE. All research personnel involved in any way with this project will have completed training in the protection of human research participants, per the guidelines issued by the U.S. Department of Health and Human Services, Office for Human Research Protection. Participants will be assigned a StudyID and only the site that enrolled each participant will have a key to match StudyID to participants name or other identifying information. Online data entry systems will be password protected and the Coordinating Center will issues password on an individual basis. Study data will

be stored on encrypted servers and accessed via password protected computers. Hard copies of study documents will be kept in locked cabinets in locked offices.

All patients may encounter the risk of inconvenience, which is RARE. We will try to avoid this by delivering surveys to patient to complete in the exam room prior to seeing the surgeon whenever possible. We will also perform functional testing in an efficient manner.

2.3.2 KNOWN POTENTIAL BENEFITS

The benefits to the study participants are minimal. All involved participants will have their distal radius fracture repaired in one of the three methods used if they had not been participating in the study.

2.3.3 ASSESSMENT OF POTENTIAL RISKS AND BENEFITS

With the exception of the risk of inconvenience of time, there are no additional risks over that of routine distal radius fracture fixation. Therefore there is very little additional risk to the study participants over what they would have assumed had they not participated in the study. The inconvenience associated with completing the study follow up measurements is outweighed by the information received about these procedures.

3 OBJECTIVES AND ENDPOINTS

OBJECTIVES	ENDPOINTS	JUSTIFICATION FOR ENDPOINTS
Primary		
To compare outcomes of DRFs in patients age 60 years and older treated with VLPS with those treated with EFP, CRPP, and casting	Michigan Hand Outcomes Questionnaire (MHQ) Summary score at 12-month assessment	The MHQ is a legacy questionnaire for assessment of hand and wrist health
Secondary		
To compare the trend of recovery for DRFs treated with VLPS with the other fixation techniques and to determine predictors of outcomes<br><br>To determine predictors of outcomes after surgical treatment of DRFs in the elderly	Michigan Hand Outcomes Questionnaire domain scores; SF-36; hand strength; wrist range of motion	MHQ domain scores allow closer inspection of specific areas of hand/wrist health; QOL and function affect outcomes and patient-reported satisfaction
Tertiary/Exploratory		
To conduct a cost-utility analysis comparing the three treatment arms with the nonsurgical arm	Medicare reimbursement; utility values derived from SF-36 scores	Most participants will be covered by Medicare; using SF-36 derived utility reduces participant burden of an additional utility questionnaire.

4 STUDY DESIGN

4.1 OVERALL DESIGN

Distal radius fractures (DRFs) are a public health concern. They represent the second most common fracture suffered by the elderly, after hip fractures.[1] In the US, approximately 10% of 65-year-old white women will suffer a DRF during their remaining lifetime, most commonly due to osteoporosis.[1] The annual direct health care expenditure on osteoporotic forearm fractures in the US is at least $385 million.[2,3] The optimal treatment strategy for DRFs in the elderly population remains controversial and undefined. Currently, there are four main types of treatment (listed in order of increasing invasiveness): close reduction and casting, percutaneous pin fixation, external fixation and internal fixation. The less invasive techniques do not require extensive dissection, but may not adequately restore anatomic alignment. This is because osteoporotic bone in the elderly may be prone to collapse and displacement when these less rigid fixation methods are used.[4,5] Recently, internal fixation techniques, namely volar plates with locking screws (VLPS), have become increasingly popular.[5-7,52] The distinct advantage of VLPS is its inherent stability. However, the internal fixation techniques require more surgical dissection and may be associated with more complications.[12] Despite these uncertainties, relatively few studies have been conducted to examine this issue. Currently available studies, mainly small trials with inadequate power, do not compare the less invasive techniques with VLPS or do not exclusively evaluate fractures in the elderly.[8-17,53-55] The main aim of this proposal is to conduct a randomized controlled clinical trial comparing VLPS to close reduction/percutaneous pinning (CRP) and external fixation±pin fixation (EFP) in treating unstable DRFs in the elderly. The primary outcome variable in this study will be the Michigan Hand Outcomes Questionnaire (MHQ).

Aim 1: To compare 24-month post-surgical outcomes of elderly DRFs treated with VLPS with CRP and EFP.
Rationale: Internal fixation allows for earlier motion of the affected hand and wrist, which prevents complications related to immobilization and potentially results in better functional outcomes.
Hypothesis: Patients treated with VLPS will have better outcomes than those treated with CRP and EFP.

Aim 2: To compare the recovery trend for elderly DRFs treated with VLPS with CRP and EFP.
Rationale: The rate of recovery for these three treatment methods is unknown. Patients will be assessed at 2 weeks, 6 weeks, 3 months, 6 months, 12 months and 24 months after surgical treatment to determine which treatment method provides the most rapid recovery.
Hypothesis: Patients treated with VLPS will have better outcomes at earlier times than those treated with the more conservative techniques, and the better outcomes will persist at the 24-month period.

Aim 3: To determine predictors of outcomes after surgical treatment of DRFs in the elderly.
Rationale: Predictors of functional outcomes in the elderly have not been studied. We will evaluate predictors of outcomes based on demographic characteristics, fracture patterns and treatment types.
Hypothesis: Severity of fracture, adequacy of reduction, bone mineral density, preinjury functional status and patient socioeconomic status are predictors of functional outcomes after treatment.

Aim 4: To compare the 12-month post-surgical outcomes and 24-month recovery trends of patients who have chosen not to have surgery with patients within the surgical arm.
Rationale: Elderly patients may have low functional demands, and it has been postulated that they can tolerate the deformity that almost certainly results following treatment with closed reduction and casting only.
Hypothesis: Patients treated with surgery will have better outcomes earlier than those treated nonsurgically.

Aim 5: To conduct a cost-utility analysis comparing the three treatment arms with the nonsurgical arm.
Rationale: A formal economic analysis comparing the three treatment options for DRFs has not been performed. We will evaluate cost and patient preferences to arrive at a dominant strategy.
Hypothesis: Although VLPS is more costly, earlier functional gains makes this treatment a dominant strategy.

4.2 SCIENTIFIC RATIONALE FOR STUDY DESIGN

There is a golden period around the introduction of a new intervention to fully evaluate its effectiveness. Once physicians have established a practice pattern, it is difficult for them to change and accept alternative treatments. Because of increasing acceptance of VLPS by hand surgeons, the appropriate time to perform a clinical trial comparing this new technology to existing technology is now.

4.3 JUSTIFICATION FOR DOSE

Not applicable.

4.4 END OF STUDY DEFINITION

A participant is considered to have completed the study if he or she has completed all phases of the study including the last visit or the last scheduled procedure shown in the Schedule of Activities (SoA), Section 1.3.

5 STUDY POPULATION

5.1 INCLUSION CRITERIA

In order to be eligible to participate in this study, an individual must meet all of the following criteria:

1. Provision of signed and dated informed consent form
2. Stated willingness to comply with all study procedures and availability for the duration of the study
3. Male or female, age 60 years and older
4. Have an unstable DRF for which surgical fixation is indicated
    a. AO type A2, A3, C1, C2

    And at least one of the following radiographic criteria indicating fracture instability

    b. Dorsal angulation of greater than -10°
    c. Radial inclination angle of less than 15°
    d. Radial shortening of greater than 3mm
5. Have the ability read, write and follow direction in English or Chinese
6. Community-dwelling patients

5.2 EXCLUSION CRITERIA

An individual who meets any of the following criteria will be excluded from participation in this study:

1. Patients who have suffered open DRFs
2. Patients with bilateral DRFs
3. Patients with associated upper extremity fractures or ligament injuries (excluding ulnar styloid fracture, TFCC and wrist ligament injuries) requiring repair at the time of DRF fixation
4. Patients with concomitant injuries that are life-threatening, require prolonged ICU stay, or require major surgical procedures
5. Patients with prior DRF to the same wrist
6. Patients with comorbid conditions prohibiting surgery
7. Patients with neurologic disorders that affect hand, wrist or arm sensation or movement
8. Patients who have a history of dementia, Alzheimer's Disease or other serious psychiatric disorders
9. Patients with current substance abuse
10. Patients who do not agree to be randomized
11. Patients who have DRFs that are not equally suited for each procedure (i.e. severely comminuted fractures)

5.3 LIFESTYLE CONSIDERATIONS

Not applicable.

5.4 SCREEN FAILURES

Screen failures are defined as participants who consent to participate in the clinical trial but are not subsequently randomly assigned to the study intervention or entered in the study. A minimal set of screen failure information is required to ensure transparent reporting of screen failure participants, to meet the Consolidated Standards of Reporting Trials (CONSORT) publishing requirements and to

respond to queries from regulatory authorities. Minimal information includes demography, screen failure details, eligibility criteria, and any serious adverse event (SAE).

5.5 STRATEGIES FOR RECRUITMENT AND RETENTION

Recruitment Plan

Participants will be recruited from patients who present to the emergency or urgent care department with a distal radius facture. A hand surgery consult will be requested. If the consulting physician or PA determines that surgical fixation will be necessary, the patient will be placed in a splint and an appointment will be scheduled at the next hand surgery clinic per institutional standard practice. When the patient is seen in the hand surgery clinic, either the hand surgeon or PA will confirm the patient's eligibility for surgery and the study and will explain the study using the provided script (MOOP Section G4). If the patient is interested in participating, a Research Coordinator will meet with the patient, explain the study further, if necessary, and obtain informed consent

Recruitment Goals: Randomized Patients

Year 1 (01/01/12-12/31/12): 14 patients
Year 2 (01/01/13-12/31/13): 14 patients (28 patients total)
Year 3 (01/01/14-06/30/14): 8 patients (36 patients total)

An addition 12 observational patients should also be recruited at a pace of 3 randomized patients to every 1 observational patient

Screening

Because emergency department flow and hand clinic scheduling vary from site to site, each site will be responsible for developing a unique system to screen and recruit eligible patients. Research personnel can expedite the recruitment process by pre-screening patents' emergency department records for mentions of prior DRFs to the same wrist, neurologic disorders affecting hand, wrist or arm sensation or movement, dementia, Alzheimer's Disease or other serious psychiatric disorders or current substance abuse. Previous medical records may be used as well. When the patient presents to clinic research personnel will assess the remaining eligibility criteria. Outcomes of eligibility screening will be recorded via the online Inclusion/Exclusion Criteria form (MOOP Section Q2).

Inclusion/Exclusion Criteria Form

For every patient who meets the initial inclusion criteria (a patient over the age of 60 years with a closed, unilateral distal radius fracture) an Inclusion/Exclusion Criteria form will be completed (MOOP Section Q2). Research staff will enter: the date the patient was screened, age, gender, race/ethnicity, eligibility status (eligible/ineligible) and participation status (eligible and enrolled / eligible and refused to provide consent). Research Coordinators should provide a brief description of the reason the patient is ineligible or why the patient refused to provide consent (if known). It should also be noted if the patient solicited advice from the surgeon as to the best treatment method, as this may be the reason for refusal to provide consent. Inclusion/Exclusion Criteria forms should be entered into REDCap on at least a bimonthly basis.

6 STUDY INTERVENTION

6.1 STUDY INTERVENTION(S) ADMINISTRATION

6.1.1 STUDY INTERVENTION DESCRIPTION

- Open reduction and internal fixation with a volar locking plating system
- Closed reduction and external fixation with a bridging fixator with or without the use of supplementary k-wire fixation
- Closed reduction and percutaneous pinning with k-wires
- Closed reduction and casting or splinting

Details of the study interventions are provided in the MOOP Section K

6.1.2 DOSING AND ADMINISTRATION

Not applicable.

6.2 PREPARATION/HANDLING/STORAGE/ACCOUNTABILITY

6.2.1 ACQUISITION AND ACCOUNTABILITY

Surgeons will use the brand and type of plates and fixators of their choice provided by their institution. Brand and type information should be recorded on the Surgery Data Form (MOOP Section Q2).

6.2.2 FORMULATION, APPEARANCE, PACKAGING, AND LABELING

Not applicable.

6.2.3 PRODUCT STORAGE AND STABILITY

Not applicable.

6.2.4 PREPARATION

Not applicable.

6.3 MEASURES TO MINIMIZE BIAS: RANDOMIZATION AND BLINDING

Randomization Plan

Randomization will be stratified by site.

Process Responsibilities

The Coordinating Center, in conjunction with Michigan Institute for Clinical and Health Research (MICHR), is responsible for maintaining the randomization website and ensuring its accuracy. The Clinical Sites are responsible for retrieving randomization assignments from the website. This includes assuring that the correct information is entered and properly recording and relaying the randomization assignment and reporting any problems experienced with the site to the Coordinating Center.

It is important to note that once a patient has been randomized they should be followed until they have completed the 24-month follow-up visit or they withdraw their consent. This applies to all patients including those that have a different procedure than the one to which they were randomized.

Procedure for Randomizing a Participant

Randomization should only be performed once all eligibility criteria have been assessed (Section 5) and the Inclusion/Exclusion form and the Mini Mental Status Exam (MOOP Section Q2) have been completed. Patients must provide informed consent form prior to randomization as well. The participant should be randomized to receive one of the three fixation techniques as soon as possible so that he/she may schedule surgery, receive pre-operative instructions and have any questions answered.

A Research Coordinator at each Clinical Site will login to the Treatment Assignment Tool – University of Michigan (TATUM) website (https://michrapps.med.umich.edu/tatum/) with their UM uniqname/username and Level 1 password (or UM Friend Account email and Level 1 password). Please enter the participant's StudyID and click "Retrieve Treatment" and "Okay" to confirm that you wish to randomize a participant. A screen will appear with the participants StudyID, Randomized ID and the Treatment Group. (See MOOP Appendix 5 for TATUM User Guide) Please record this information for entry in REDCap in the Study Arm form. An automatically generated email will be sent to the Coordinating Center and to the Clinical Site. Once the randomization assignment has been retrieved, the researcher will notify the surgery scheduler and record this information in the participant's study file.

The surgery scheduler should make every attempt to schedule the procedure without the surgeon knowing the exact procedure to be performed. Methods for doing this will vary from site to site; at the University of Michigan this will be achieved by scheduling the procedure as a "WRIST distal radius fixation." Surgical staff will prepare materials for each of the three procedures. No more than 24 hours before the procedure the surgeon should be informed of which procedure the patient was randomized to. This should be done using 2 methods, in case one method fails. These methods may include presenting an envelope to the surgeon, email, text message, or contacting the surgery scheduler to reveal the procedure in the schedule.

6.4 STUDY INTERVENTION COMPLIANCE

The PI is responsible for informing the participant of the procedure to which he/she was randomized and should be available to answer any questions the participant may have.

Each participant should receive the procedure to which her/she was randomized. In the event that the participant does not receive the randomized procedure, for whatever reason, the Protocol Deviation Form (MOOP Section Q2) should be completed and the Coordinating Center should be notified within 48 hours.

As soon as possible following surgery, the PI should complete the Surgery Data Form (MOOP Section Q2). Data should be derived from OR or anesthesiology records for accuracy and SHOULD NOT be estimated.

6.5 CONCOMITANT THERAPY

Not applicable.

6.5.1 RESCUE MEDICINE

Not applicable.

7 STUDY INTERVENTION DISCONTINUATION AND PARTICIPANT DISCONTINUATION/WITHDRAWAL

7.1 DISCONTINUATION OF STUDY INTERVENTION

Participant cross-over, for any reason, should be reported to the Coordinating Center in the same manner. The Protocol Deviation log (MOOP Section Q2) should be completed and the Coordinating Center should be notified within 24 hours.

Cross-over does not mean discontinuation from the study, and remaining study assessment should be completed as indicated by the study protocol.

7.2 PARTICIPANT DISCONTINUATION/WITHDRAWAL FROM THE STUDY

Participants are free to withdraw from participation in the study at any time upon request.
An investigator may discontinue or withdraw a participant from the study for the following reasons:

- If any clinical adverse event (AE), laboratory abnormality, or other medical condition or situation occurs such that continued participation in the study would not be in the best interest of the participant
- New disease or condition or worsening of existing disease or condition that makes it impossible for the participants to complete follow-up assessments (e.g. participant enters hospice/comfort care)

The reason for participant discontinuation or withdrawal from the study should be communicated to the Coordinating Center as soon as possible.

7.3 LOST TO FOLLOW-UP

A participant will be considered lost to follow-up if he or she fails to return for 24-month follow-up and is unable to be contacted by the study site staff.

The following actions must be taken if a participant fails to return to the clinic for a required study visit:

- The site will attempt to contact the participant and reschedule the missed visit within the study window (Section 5.5) and counsel the participant on the importance of maintaining the assigned visit schedule and ascertain if the participant wishes to and/or should continue in the study.
- Before a participant is deemed lost to follow-up, the investigator or designee will make every effort to regain contact with the participant including phone, mail, email, or communication through EMR.
- If the participant is willing to continue participation, but will not agree to return to clinic study surveys can be completed by mail, email, or phone.
- Should the participant continue to be unreachable, he or she will be entered as terminated with a primary reason of lost to follow-up.

8 STUDY ASSESSMENTS AND PROCEDURES

8.1 EFFICACY ASSESSMENTS

8.1.1 TIMELINE AND SCHEDULE OF VISITS

See section 1.3 Schedule of Activities

8.1.2 SCREENING EVALUATION

Screening

For every patient who meets the initial inclusion criteria (a patient over the age of 60 years with a closed, unilateral distal radius fracture) an Inclusion/Exclusion Criteria form will be completed (MOOP Section Q2). Research staff will enter: the date the patient was screened, age, gender, race/ethnicity, eligibility status (eligible/ineligible) and participation status (eligible and enrolled / eligible and refused to provide consent). Research Coordinators should provide a brief description of the reason the patient is ineligible or why the patient refused to provide consent (if known). It should also be noted if the patient solicited advice from the surgeon as to the best treatment method, as this may be the reason for refusal to provide consent. Inclusion/Exclusion Criteria forms should be entered into REDCap on at least a bimonthly basis.

Screening Period

Radiographic inclusion criteria may be evaluated pre- or post-reduction. Patients who meet all inclusion/exclusion criteria, including radiographic criteria, pre-reduction may be eligible for the study in two manners. Patients whose post-reduction x-rays indicate unacceptable reduction are eligible immediately. Patients whose post-reduction x-rays indicate acceptable reduction are eligible if x-rays indicate an unacceptable reduction

During screening, patient's eligibility will be assessed based on the inclusion and exclusion criteria as described in Sections 5.1 and 5.2.

8.1.3 ENROLLMENT AND BASELINE ASSESSMENTS

Enrollment

Our enrollment date is when the screening criteria are met and informed consent is signed by the patient.

Consenting Procedure

This study has a single informed consent form that describes both the screening and study procedures.

The Research Coordinator at each site will explain the study, review study requirements and emphasize the voluntary nature of participation to potential participants in detail. Additionally, the patient will also be told, as stated in the informed consent document, that non-participation will not affect his/her medical care in any way. The Research Coordinator will briefly detail the casting and other three procedures, in plain language, and explain that the procedures may be equally affective at repairing his/her distal radius fracture and that there is no "best" treatment. The Research Coordinator will briefly explain the randomization process and follow-up measures. Finally, the Research Coordinator will tell the patient that he/she will be compensated $20 per follow-up assessment he/she completes, for a total of $140.

A written comprehensive informed consent document will be used. Each institution will use its own informed consent document and follow its own policies to obtain informed consent. Once the patient has fully read the informed consent document and all concerns have been addressed, the patient may sign the document. Each participant will also receive a copy of the informed consent document for his/her records.

The signed informed consent form should be kept by each Clinical Site in their locked participant study files for a period of at least three years or longer if required by the Clinical Site's IRB. Copies should be transmitted to the Coordinating Center via email, fax or mail on at least a monthly basis.

Baseline Assessments

X-ray: An X-ray of the patient's fractured arm will be done.

Mini Mental State Examination: For this exam the following supplies are needed: a blanks sheet of paper, a note card with the words "Close your eyes" printed clearly and a pen. The participant should be seated and asked to perform the various tasks that comprise the MMSE. A score will be calculated immediately. Patients with a score of 24 or below are considered ineligible

Participant Demographics: Data collected will include date of birth, gender, hand dominance, income and occupation. Injury data will include date of injury, date of surgery, injured arm, AO classification of

fracture, mechanism of injury, associated injuries, and any pre-existing upper extremity trauma or impairment.

Comorbidity checklist: It is a self-administered comorbidity questionnaire that the patient answers and will include information on patient's medical conditions such as heart disease, diabetes, or other medical problems.

RAPA: Rapid Assessment of Physical activity is a questionnaire about the amount and intensity of physical activity patients usually do. The RAPA is a 9 question survey that has been validated for use in adults over 50 years of age and has shown better sensitivity and specificity than other frequently used assessments of physical activity.

SF-36: Short Form (36) is a survey of patients' health to assess their health status.

Pain Domain of MHQ: A questionnaire to assess the amount of pain the patients had in their hand or wrist during the past week.

Participant payment: upon completion of consenting and enrollment the study participant will be paid $20.

Adverse event and protocol deviation information will be collected if necessary during the visit.

8.1.4 FOLLOW-UP ASSESSMENTS

Visit 3: Scheduled at 2 weeks after the surgery or closed reduction

- X-Ray
- SF-36
- General Health Questionnaire
- Fingertip to palmar Crease Distance Form
- Complication Checklist
- Hand Therapy Data form
- Pain Domain of MHQ
- Hand function Data Form
- Participant payment
- Adverse Event collection
- Protocol deviation collection

Visit 4: Scheduled at 6 weeks following surgery or closed reduction

- X-Ray
- SF-36
- General Health Questionnaire
- Fingertip to palmar Crease Distance Form
- Complication Checklist
- Hand Therapy Data form
- MHQ(all domains)
- Hand function Data Form
- Participant payment

- Adverse Event collection
- Protocol deviation collection

<u>Visit 5:</u> Scheduled at 3months following surgery or closed reduction
<u>Visit 6:</u> Scheduled at 6 months following surgery or closed reduction
<u>Visit 7:</u> Scheduled at 12 months following surgery or closed reduction
<u>Visit 8:</u> Scheduled at 24 months following surgery or closed reduction (additional assessment of RAPA will be done at this visit)

- X-Ray
- SF-36
- General Health Questionnaire
- Complication Checklist
- Hand Therapy Data form
- MHQ(all domains)
- Hand function Data Form
- Participant payment
- Adverse Event collection
- Protocol deviation collection

8.1.5 FINAL EVALUATION

The 8th visit scheduled at 24 weeks following surgery or closed reduction is the final evaluation visit.

If the participant is not able to be evaluated within the appropriate time period, the reason should be noted in the comments section of the participant appointment log and the participant is considered lost to follow-up for that visit. If possible, the MHQ, SF-36 and RAPA, along with a postage paid envelope, should be sent to the participant. Despite missing one follow-up visit, the participant is still considered to be actively enrolled in the study and should be followed up for future visits.

Each participant should be reminded at the end of every visit when he/she will need to be seen again (i.e. “We’ll see you in 6 months.”) and should schedule an appointment, if possible. If a participant has not made a clinic appointment within 2 weeks of his/her follow-up date, a member of the research team should contact the participant via phone to schedule an appointment. The participant should be reminded that he/she is a valuable member of the research team and that participation is very important. A participant is considered to be active and should be reminded to return for follow-up assessment unless he/she chooses to withdraw from the study.

In an effort to collected as much data as possible, participants who were enrolled in the study at the time of their 24-month assessment and who did not complete the assessment will be contacted at the end of the study (Early 2019). Traditional 24 month surveys (MHQ, SF-36, and RAPA) will be sent to participants via postal mail.

8.2 SAFETY AND OTHER ASSESSMENTS

Not applicable.

8.3 ADVERSE EVENTS AND SERIOUS ADVERSE EVENTS

8.3.1 DEFINITION OF ADVERSE EVENTS (AE)

The NIH defines an AE as any unfavorable and unintended diagnosis, symptom, sign (including an abnormal laboratory finding), syndrome, or disease, which may or may not be related to the intervention. AES include any new events not present during the pre-intervention period or events that were present during the pre-intervention period which has increased in severity. Sites should also check their IRB's for site-specific definitions and further guidance.

8.3.2 DEFINITION OF SERIOUS ADVERSE EVENTS (SAE)

The NIH defines an SAE as any untoward medical occurrence that results in death, is life-threatening, requires or prolongs hospitalization, causes persistent or significant disability/incapacity, results in congenital anomalies/birth defects, or, in the opinion of the investigators, represents other significant hazards or potentially serious harm to research participants or others. Sites should also check their IRB's for site-specific definitions and further guidance.

8.3.3 CLASSIFICATION OF AN ADVERSE EVENT

8.3.3.1 SEVERITY OF EVENT

For adverse events (AEs) the following guidelines will be used to describe severity.

- **Mild** – symptoms, no specific treatment
- **Moderate** – diagnostic procedure, specific diagnosis, and/or nonsurgical treatment
- **Severe** – surgical treatment required

8.3.3.2 RELATIONSHIP TO STUDY INTERVENTION

Any of the above events can be considered Related or Unrelated. An event is Related if there is a reasonable possibility that the event may have been caused by or is linked in a significant way to the research. This encompasses all aspects of the research—it is not limited to test agents or procedures.

Unrelated (clearly not related to the research)

Unlikely (doubtfully related to the research)

Probable (likely related to the research)

Definite (clearly related to the research)

8.3.3.3 EXPECTEDNESS

Unexpected Adverse Event: An unexpected adverse event is an adverse reaction, the nature or severity of which is not consistent with the applicable product information (e.g., package insert/summary of product characteristics for an approved product or device).

8.3.4 TIME PERIOD AND FREQUENCY FOR EVENT ASSESSMENT AND FOLLOW-UP

The follow up plan for AEs will be AE-specific and so is the duration of the follow-up.

8.3.5 ADVERSE EVENT REPORTING

The PI(s) at each site are responsible for making determinations regarding adverse events with respect to severity and/or attribution. However all members of the research team are responsible for reporting possible AEs to the PIs. All adverse events and unexpected adverse events should be reported to the clinical site's IRB per institutional standards. Adverse events will be reported to the coordinating center upon request to facilitate semi-annual reporting to NIAMS and the DSMB (through KAI).

8.3.6 SERIOUS ADVERSE EVENT REPORTING

The PI(s) at each site are responsible for making determinations regarding adverse events with respect to severity and/or attribution. However all members of the research team are responsible for reporting possible AEs to the PIs. Serious adverse events should be reported to the coordinating center. Clinical sites will also report events to their IRB, per institutional standards. The coordinating center will report events to University of Michigan IRBMED, NIAMS and the DSMB (through the executive secretary, KAI research, Inc.) within 48hus of the investigator becoming aware of the event.

8.3.7 REPORTING EVENTS TO PARTICIPANTS

Not applicable.

8.3.8 EVENTS OF SPECIAL INTEREST

Not applicable.

8.3.9 REPORTING OF PREGNANCY

Not applicable.

9 STATISTICAL CONSIDERATIONS

9.1 STATISTICAL HYPOTHESES

Aim 1: To compare 24-month post-surgical outcomes of elderly DRFs treated with VLPS with CRP and EFP.
Rationale: Internal fixation allows for earlier motion of the affected hand and wrist, which prevents complications related to immobilization and potentially results in better functional outcomes.
Hypothesis: Patients treated with VLPS will have better outcomes than those treated with CRP and EFP.

Aim 2: To compare the recovery trend for elderly DRFs treated with VLPS with CRP and EFP.
Rationale: The rate of recovery for these three treatment methods is unknown. Patients will be assessed at 2 weeks, 6 weeks, 3 months, 6 months, 12 months and 24 months after surgical treatment to determine which treatment method provides the most rapid recovery.
Hypothesis: Patients treated with VLPS will have better outcomes at earlier times than those treated with the more conservative techniques, and the better outcomes will persist at the 24-month period.

Aim 3: To determine predictors of outcomes after surgical treatment of DRFs in the elderly.
Rationale: Predictors of functional outcomes in the elderly have not been studied. We will evaluate predictors of outcomes based on demographic characteristics, fracture patterns and treatment types.
Hypothesis: Severity of fracture, adequacy of reduction, bone mineral density, preinjury functional status and patient socioeconomic status are predictors of functional outcomes after treatment.

Aim 4: To compare the 12-month post-surgical outcomes and 24-month recovery trends of patients who have chosen not to have surgery with patients within the surgical arm.
Rationale: Elderly patients may have low functional demands, and it has been postulated that they can tolerate the deformity that almost certainly results following treatment with closed reduction and casting only.
Hypothesis: Patients treated with surgery will have better outcomes earlier than those treated nonsurgically.

Aim 5: To conduct a cost-utility analysis comparing the three treatment arms with the nonsurgical arm.
Rationale: A formal economic analysis comparing the three treatment options for DRFs has not been performed. We will evaluate cost and patient preferences to arrive at a dominant strategy.
Hypothesis: Although VLPS is more costly, earlier functional gains makes this treatment a dominant strategy.

9.2 SAMPLE SIZE DETERMINATION

We propose to randomize 474 patients to one of three surgical arms (158 per surgical arm). We will follow another 158 non-surgical patients meeting the inclusion/exclusion criteria. Sample size was determined to have adequate power for Aim 1 based on the primary outcome of the MHQ summary score at 12-month follow-up. **We expect to have complete data for 12-month follow-up time from 126 patients in each group, after accounting for a conservatively estimated 20% decrease in sample size from losses to follow-up.** For Aim 1, we expect 126 patients per group to give 80% power to detect an 8 point difference in the mean MHQ summary scores between VLPS and CRP and between VLPS and EFP using a mixed-effects model with a two-sided 0.025 α-level test. This assumes a standard deviation (SD) of 18 points (corresponding to an effect size of 0.44 SD), an average of 24 patients per site and an intra-site correlation coefficient (ICC) of 0.012**. To adjust for potential within surgeon correlation, we will also consider either a three level hierarchical model with surgeons nested within site, or use surgeons instead of sites as random intercepts. If technical variation is mostly between surgeons, addressing within surgeon correlation will reduce the ICC, which would give larger power to detect the same effect size. Covariate adjustments will likely give greater power by reducing the variability when the covariates are associated with the outcome and are not highly correlated with other covariates in the model.** We expect the 8 point difference in MHQ score at 12-months as a minimally detectable and meaningful difference between VLPS and either CRP or EFP.[56] Our retrospective study of patients treated with VLPS showed mean (±SD) MHQ scores of 77 (±15) at 3 months, 81 (±17) at 6 months, and 85 (±18) at 12 months for elderly patients.[42] We believe the observed improvement of 8 points from 3 to 12 months to be similar to the expected post-surgical improvement difference between VLPS surgery compared with either EFP or CRP.

For Aim 2, the proposed sample size and longitudinally assessed outcomes will provide 80% power to detect a between-group difference in the monthly rates of improvement of 0.22 points or greater with an α level of 0.025, assuming a within-person correlation of 0.2 and ICC of 0.012. **For Aim 3, we consider power for a hypothetical scenario in which VLPS effect is smallest in the least active and largest in the most active patients. The study will have 83% power to detect the difference in the effect sizes for the VLPS to CRP group of 0.05 SD in the lowest, 0.30 SD in the moderate and 1.0 SD in the highest activity subgroups based on a 2 by 3 two-way analysis of variance, assuming about even number of patients in the three baseline activity subgroups. We note that though we do not hypothesize a differential effect of treatment in any subgroup, we will have adequate power to detect a differential treatment effect as large or larger than the hypothesized scenario, and the study will have adequate power to detect predictor effects that are homogeneous across subgroups as long as the predictor effects are as large as the effect size associated with the surgical procedures.** Aim 4 will compare outcomes between the combined surgical groups versus non-surgical group in which we expect the difference to be larger than the differences between VLPS and either EFP or CRP. We therefore expect the statistical power using the proposed number of non-surgical group patients to be more than adequate to make this comparison.

As described in Section C11 under Aim 1, a small portion of patients may cross over intra-operatively from a randomly assigned procedure of EFP or CRP to VLPS. Whether the analysis is done using intent-to-treat or as-treated, such cross-over cases may result in somewhat smaller effect sizes than when there were no cross-over cases. Nonetheless, having outcomes measured longitudinally over a 24-month period gives the study adequate power to detect a time-averaged difference between two surgical arms much smaller than the difference based only at 12 months. Specifically, the proposed study gives 80% power to detect a time-averaged difference as small as 4.8 points, using a 0.025 level two-sided test based on mixed-effects model with three follow-up assessments and assuming an SD of 18 and a within-person correlation of 0.2. In addition, using the as-treated analysis, in a worst case scenario in which 10% of both EFP and CRP patients cross over to VLPS, sample size will not be equal across the three groups. This, however, will not affect the power significantly. Specifically, we will still have 80% power to detect a time-averaged difference in the MHQ scores of about 5 points between EFP or CRP (N = 113 per group) to VLPS (N = 152) with 0.025 level test. The power calculations were done using NQuery Advisor 7.0 (Saugus, MA), with appropriate considerations for longitudinally measured data.[57]

9.3 POPULATIONS FOR ANALYSES

The primary analytical approach will be intention-to-treat. An as-treated approach will be used for sensitivity analyses.

9.4 STATISTICAL ANALYSES

9.4.1 GENERAL APPROACH

The study design chosen is a Randomized Controlled Trial (RCT), because RCT's provide the most reliable form of scientific evidence. Few retrospective case control studies were conducted so far regarding distal radius fractures. A multicenter randomized clinical trial to define the optimal treatment for this prevalent injury in the elderly was not conducted so far.

9.4.2 ANALYSIS OF THE PRIMARY EFFICACY ENDPOINT(S)

Aim 1: *To compare 12-month post-surgical outcomes of elderly DRFs treated with VLPS with CRP and EFP.* Primary outcome will be the summary score of the MHQ. We will report means for each group and the mean difference between pairs of each group, along with their 95% confidence intervals. The primary outcome will be compared using a linear mixed-effects model[58] to address the hierarchical nature of the data. The model will include surgeons nested within site and sites as random effects, fracture severity group indicator effect and two indicators for EFP and CRP groups. The surgical procedure indicators will each estimate the difference between EFP to VLPS and CRP to VLPS. We will also obtain covariate adjusted estimates of the between group differences; the covariates will be determined by the baseline analyses and will likely include age, baseline MHQ score, dominance of the injured hand and baseline RAPA score. Similar analyses will be done using other secondary outcome variables, including grip strength. We will also calculate MHQ score and grip strength as the difference (and %) of the uninjured hand scores. These measures may better reflect recovery because the uninjured hand can give a proxy measure of the patient-specific maximum achievable outcome. Analysis of complications will depend on how often they occur. For example, if the combined complications are rare, we will dichotomize them as present/absent and use a generalized linear mixed-effects model with logit link to compare the odds of complication between VLPS/EFP and between VLPS/CRP, while adjusting for within surgeon and site correlation. If the number of complications is substantial, we will use a count data model such as generalized linear model with log link to make the between-group comparison.

Cross-over: A surgeon may decide intra-operatively that the randomly assigned procedure is not amenable to the patient's particular fracture, which results in an intra-operative cross-over. Due to the nature of the surgery, such intra-operative cross-over cases will be limited to crossing from either of the two more conservative procedures (EFP or CRP) to VLPS, and we expect it to occur in < 5% of the cases. Intent-to-treat (ITT) will be the primary analytic method with participants retained in their randomized groups regardless of whether they have crossed over. The ITT estimates the use effectiveness and is the standard way to handle cross-over in randomized controlled trials[29] and has been used in other DRF fixation studies.[10,11,16,17,59] We will also do as-treated (AT) analyses in which groups will be defined by the procedure the patient ultimately receive. In this study, cross-over will likely give an under-estimate of the VLPS effect (compare with either EFP or CRP) with both ITT and AT analyses. This is because any cross-over will be to VLPS, the procedure expected to give better outcomes. With ITT analysis, the mean difference between VLPS and either EFP or CRP will likely be smaller because those who crossed to VLPS

(who would be expected to have better outcomes) will be analyzed as EFP or CRP. In surgical trials, another potential form of cross-over is a possible repeat surgery (of the same or different type) due to complication or failure to achieve acceptable reduction. In elderly DRF patients, most surgeons will not recommend another surgery due to less than desirable reduction. This is because there has been no evidence that failure to achieve reduction affects functional recovery.[60,61] Even if a repeat surgery is desired, it will not be within a year from the initial surgery when functional problems occur.

Missing Data: We will describe the extent and pattern of missingness in outcome variables by the three groups. Although not expected to be more than 2-3%, we will assess the amount of missing data in baseline covariates as well. If missingness is less than 5% and missing data appear to be at random (e.g., depends on covariates, but not suspected to depend on unobserved outcome) or completely random, we will fit a model using complete data, adjusting for the covariates that missingness is found to depend on. On the other hand, if missingness is greater than 5%, we will impute the missing outcome values based on baseline prognostic factors, and 6-week and 3-month outcome values. Multiple imputation will be implemented using IVEware software, which was developed at the University of Michigan and uses a multivariate sequential regression approach.[62] IVEware can impute all types of data (binary, categorical, count and continuous) and imputes missing values for each individual conditional on all observed values for that individual, and thus exploits correlational structure among covariates that include all other variables observed or imputed. We will then obtain parameter estimates of interest using the described analytic approach in which we will combine the estimates from the five imputed datasets, accounting for both within and between imputation variability.[63]

Aim 2: *To compare the recovery trend for elderly DRFs treated with VLPS with CRP and EFP.* This aim is to provide an understanding of the recovery pace and long-term outcomes after DRF and subsequent treatment. We think patients will show steady improvement and will eventually reach a plateau in their outcomes, but we currently do not know how long it takes to reach the plateau and if significant differences in improvement occur in early months. We will first graphically explore the outcomes data over time by plotting cross-sectional means at each measurement time by surgical group and by plotting longitudinal outcomes over time for each individual to assess individual trends. We will use a longitudinal data mixed-effects model to assess and compare outcome trends over time within and across the three groups.[58] The model will estimate the time-averaged outcomes for the three groups, the average slope over time in the VLPS group and the effect of EFP and CRP on the slope of the VLPS group. The EFP effect and the CRP effect on the average slope of the VLPS group is each hypothesized to be negative because we hypothesize the recovery rate of each to be slower than that of VLPS. We will use an autoregressive covariance specification first, but appropriateness of other covariance specification will also be considered. The graphical exploration will help us consider more complicated parameter structures of the mixed-effects models describing the trajectory of recovery, especially how the time and the correlation within patients will be modeled. This analysis will allow us to assess when the outcome differences occur and whether the differences remain over time using various ways to model time. In addition to comparing the rates of improvement over time, we will compare the outcome differences at 24 months.[64] Similar analyses will be done using other secondary outcome

variables, such as grip strength and pain. As with Aim 1, we will model trends using the intention-to-treat and as-treated method.

9.4.3 ANALYSIS OF THE SECONDARY ENDPOINT(S)

Aim 3: *To determine predictors of outcomes after surgical treatment of DRFs in the elderly.* We will assess the effect of potential predictors of outcomes within each surgical treatment group and assess whether the effect remains the same across the treatment groups. Potential predictors are variables found to be associated with post-surgical outcomes in our previous studies and include fracture severity[65], reduction adequacy (measured, for example, using intra-articular step-off for intra-articular fractures), age, income level, baseline activity level and bone mineral density. The analysis will initially be done separately in the three surgical groups using the linear mixed-effects models and 12-month outcomes. Other variables potentially associated with outcome variation are pre-existing arthritis, extensive soft-tissue injury, presence of nerve injury, presence of other associated injuries and whether the dominant hand is injured. Although some of the proposed potential predictors are likely to be collinear, we do not have two variables that are highly collinear on face value. We will, however, assess multi-collinearity and employ data reduction strategies to reduce the number of variables. In a separate model, we will also consider 6-week values of the outcome variable as a potential predictor of long-term outcome. Within each surgical group, other variables will be considered such as an indicator for additional fixation requirement using additional plates or screws as a part of the VLPS placement, because the added fixation procedures may indicate a more severe fracture pattern. For continuous variables such as age, we will explore a proper functional relationship using age categorical indicators and splines. If the models for each surgical group show similar relationships between predictors and outcome variables, we will combine the data across the surgical groups. We do not have a hypothesized differential effect of a predictor on outcomes that depend on surgical group, and thus interactions will be explored only if a large difference in the relationship between a predictor and outcomes are observed between any two surgical groups. Similar analyses will be done using secondary outcome variables of interest.

Aim 4: *To compare the 12-month post-surgical outcomes and 24-month recovery trends of patients who have chosen not to have surgery with patients within the surgical arm.* Many elderly patients have low functional demands and can tolerate the deformity that almost certainly results following closed reduction.[53,66-68] Therefore we will compare the long-term outcomes and the trends between the surgical and non-surgical groups. For a valid comparison, it will be essential to account for baseline differences that would potentially result in treatment choices and outcome differences. This will be done using propensity score (PS) method and the inverse-probability treatment weighted (IPTW) method.[69] The goal of both methods is to estimate the outcome differences between the surgical and non-surgical groups at 12 months, adjusting for selection bias using the measured potential confounding variables. In the PS method, we will calculate the propensity for choosing to have surgery, evaluate the distribution of the estimated propensity scores and perform the comparison stratified by the propensity quintiles. We will estimate the propensity scores using a logistic regression model with surgical group as the dependent variable. The propensity model will not be parsimonious and will include as predictors all

demographic and other baseline variables, in particular those representing preference toward choosing surgery such as physical activity level prior to fracture. The stratified comparison between the surgical groups combined and the non-surgical group will be done using a linear mixed-effects model[58] with the 12-month MHQ score as the dependent variable and the propensity quintiles as the strata, after trimming. The model will include site as random-effects to adjust for within-site correlation and an indicator for surgical groups; the parameter estimate for the indicator will estimate the outcome difference between the surgical and non-surgical groups. For the IPTW method, a similar linear mixed-effects model will be fit, but using the inverse of the estimated probability-of-treatment as weights in which the probability-of-treatment is estimated from the propensity model. The linear mixed-effects model can still include baseline covariates that may affect the outcome differences. Lastly, we will visualize the outcome trends between the four groups over the 24-month period and model the long-term differences. The longitudinal outcome model will also include time and an interaction of time by the surgical groups. If the trends appear to differ across all four groups, richer parameterization will be done as needed; the analytic approach for trend comparison in hand outcomes during follow-up will generally be similar to the Aim 2 analytic plan.

Aim 5: To conduct a cost-utility analysis comparing the three treatment arms with the nonsurgical arm. To calculate QALYs for a cost-utility analysis, we have already begun development and initial testing of an online time trade-off (TTO) survey to determine utility for various health states related to DRF. Utility is the preference assigned to a particular health state on a scale where 0 represents death and 1 represents perfect health. Using the TTO method, a person is asked to choose how much time in his/her life he/she is willing to give up to be in a healthier state while avoiding a less healthy one. We will assess the utilities for different health states that could occur (malunion, nonunion, infection, etc) following DRF treatment. Data from the current study will be used to determine the outcomes and complications of DRFs. QALYs will then be calculated from the utilities, adjusted for years of life remaining for different age groups. We will also obtain utilities of elderly subjects from the community for a comprehensive analysis of the societal perspective, as recommended by the Panel on Cost Effectiveness in Health and Medicine.[70,71] Medical costs will be assessed using the relevant charges/cost data for the procedures on all participants recruited at the Michigan Medicine. The costs of lost productivity will be valued as the average US hourly wage, as obtained from the US Bureau of Labor Statistics, of a person most likely to be affected by a DRF, multiplied by the average time to healing, as obtained from this study. Costs will be discounted at a rate of 3%.[44,45] Sensitivity analyses will be done to change the assumptions to fit the extremes of these estimates. If, in changing these estimates, the results are similar, we can be assured of the stability of the conclusions. One- and two-way sensitivity analyses will be performed to determine the stability of the incremental cost-utility ratio, which will incorporate the cost and utility range of the study subjects' in the calculations

9.4.4 SAFETY ANALYSES

Triggers for ad hoc review

Serious Adverse events that result in participant death will trigger ad hoc review, which will take place between the DSMB, the Coordinating Center and the involved Performance Site via conference call

Stopping Rules

Human subjects recruitment will cease if the University of Michigan IRB or NIAMS, based on the recommendation from the DSMB, determine that there is an unacceptable number of adverse events, if the serious, related adverse event rates differ between the two study groups at an alpha level of 0.05 at each time when side effects are assessed for DSMB report, or if it becomes apparent that during the planned 2.5 years of participant recruitment, we will achieve less than 25% of our recruitment goal.

9.4.5 BASELINE DESCRIPTIVE STATISTICS

We will compare the three groups to ensure reasonable similarity in their baseline characteristics using analysis of variance for continuous variables such as age, and chi-square test for categorical variables such as sex. Because the sample size is fairly large, we do not expect clinically meaningful baseline imbalances, but we will include baseline variables showing differences across the groups with $p< 0.15$ as potential covariates in the primary analysis.

9.4.6 PLANNED INTERIM ANALYSES

Not applicable.

9.4.7 SUB-GROUP ANALYSES

Aim 3: *To determine predictors of outcomes after surgical treatment of DRFs in the elderly.* We will assess the effect of potential predictors of outcomes within each surgical treatment group and assess whether the effect remains the same across the treatment groups. Potential predictors are variables found to be associated with post-surgical outcomes in our previous studies and include fracture severity[65], reduction adequacy (measured, for example, using intra-articular step-off for intra-articular fractures), age, income level, baseline activity level and bone mineral density. The analysis will initially be done separately in the three surgical groups using the linear mixed-effects models and 12-month outcomes. Other variables potentially associated with outcome variation are pre-existing arthritis, extensive soft-tissue injury, presence of nerve injury, presence of other associated injuries and whether the dominant hand is injured. Although some of the proposed potential predictors are likely to be collinear, we do not have two variables that are highly collinear on face value. We will, however, assess multi-collinearity and employ data reduction strategies to reduce the number of variables. In a separate model, we will also consider 6-week values of the outcome variable as a potential predictor of long-term outcome. Within each surgical group, other variables will be considered such as an indicator for additional fixation requirement using additional plates or screws as a part of the VLPS placement, because the added fixation procedures may indicate a more severe fracture pattern. For continuous

variables such as age, we will explore a proper functional relationship using age categorical indicators and splines. If the models for each surgical group show similar relationships between predictors and outcome variables, we will combine the data across the surgical groups. We do not have a hypothesized differential effect of a predictor on outcomes that depend on surgical group, and thus interactions will be explored only if a large difference in the relationship between a predictor and outcomes are observed between any two surgical groups. Similar analyses will be done using secondary outcome variables of interest.

9.4.8 TABULATION OF INDIVIDUAL PARTICIPANT DATA

Not applicable.

9.4.9 EXPLORATORY ANALYSES

Aim 5: To conduct a cost-utility analysis comparing the three treatment arms with the nonsurgical arm. To calculate QALYs for a cost-utility analysis, we have already begun development and initial testing of an online time trade-off (TTO) survey to determine utility for various health states related to DRF. Utility is the preference assigned to a particular health state on a scale where 0 represents death and 1 represents perfect health. Using the TTO method, a person is asked to choose how much time in his/her life he/she is willing to give up to be in a healthier state while avoiding a less healthy one. We will assess the utilities for different health states that could occur (malunion, nonunion, infection, etc) following DRF treatment. Data from the current study will be used to determine the outcomes and complications of DRFs. QALYs will then be calculated from the utilities, adjusted for years of life remaining for different age groups. We will also obtain utilities of elderly subjects from the community for a comprehensive analysis of the societal perspective, as recommended by the Panel on Cost Effectiveness in Health and Medicine.[70,71] Medical costs will be assessed using the relevant charges/cost data for the procedures on all participants recruited at the Michigan Medicine . The costs of lost productivity will be valued as the average US hourly wage, as obtained from the US Bureau of Labor Statistics, of a person most likely to be affected by a DRF, multiplied by the average time to healing, as obtained from this study. Costs will be discounted at a rate of 3%.[44,45] Sensitivity analyses will be done to change the assumptions to fit the extremes of these estimates. If, in changing these estimates, the results are similar, we can be assured of the stability of the conclusions. One- and two-way sensitivity analyses will be performed to determine the stability of the incremental cost-utility ratio, which will incorporate the cost and utility range of the study subjects' in the calculations

10 SUPPORTING DOCUMENTATION AND OPERATIONAL CONSIDERATIONS

10.1 REGULATORY, ETHICAL, AND STUDY OVERSIGHT CONSIDERATIONS

10.1.1 INFORMED CONSENT PROCESS

10.1.1.1 CONSENT/ASSENT AND OTHER INFORMATIONAL DOCUMENTS PROVIDED TO PARTICIPANTS

Each institution should use the informed consent document approved by their IRB.

10.1.1.2 CONSENT PROCEDURES AND DOCUMENTATION

Once an eligible, interested patient has been identified a Research Coordinator, surgeon, or PA will approach the patient to discuss the study. The Research Coordinator at each site will explain the study, review study requirements and emphasize the voluntary nature of participation to potential participants in detail. Additionally, the patient will also be told, as stated in the informed consent document, that non-participation will not affect his/her medical care in any way. The Research Coordinator will briefly detail casting and the three surgical procedures, in plain language, and explain that the procedures may be equally affective at repairing his/her distal radius fracture and that there is no "best" treatment. The Research Coordinator will briefly explain the randomization process and follow-up measures. Finally, the Research Coordinator will tell the patient that he/she will be compensated $20 per follow-up assessment he/she completes, for a total of $140.

A written comprehensive informed consent document will be used. Different informed consent documents may be used for the randomized, surgical group and the non-randomized, non-surgical group. Each institution will use its own informed consent document and follow its own policies to obtain informed consent. Once the patient has fully read the informed consent document and all concerns have been addressed, the patient may sign the document. Each participant will also receive a copy of the informed consent document for his/her records.

The signed informed consent form should be kept by each Clinical Site in their locked participant study files for a period of at least three years or longer if required by the Clinical Site's IRB. Copies should be transmitted to the Coordinating Center via email, fax or mail on at least a monthly basis.

10.1.1.3 STUDY DISCONTINUATION AND CLOSURE

Human subjects recruitment will cease if the University of Michigan IRB or NIAMS, based on the recommendation from the DSMB, determine that there is an unacceptable number of adverse events, if the serious, related adverse event rates differ between the two study groups at an alpha level of 0.05 at each time when side effects are assessed for DSMB report, or if it becomes apparent that during the planned 2.5 years of participant recruitment, we will achieve less than 25% of our recruitment goal.

10.1.2 CONFIDENTIALITY AND PRIVACY

The following is a list of participant confidentiality safeguards:

- Participant identifying information such as names or medical identification numbers are not to be included on hard copies of the data forms.
- Participant identifying information stored electronically must be maintained in a database separate from other information and maintained in a secure location.
- Forms or pages containing identifying information should be separated from other pages of the data forms.
- Data listings that contain participant name, medical identification number, or other identifiers easily associated with a specific participant should not be distributed.
- Participant name, medical identification number, or other unique identifiers, such as Social Security number, should not be included in any published data listing.
- Participant records stored in the data center should not be accessible to persons outside the center without the express written consent of the participant.
- Study forms and related documents retained both during and after study completion should be stored in a secure location.

Computer Data Storage:

- Passwords should be used and changed on a regular basis.
- Study staff that has access to clinical computer systems should be trained in their use and in related security measures.
- Prior to the use of a new computer system, and if it is modified, the system should be tested to verify that it performs as expected.
- Backup copies of electronic data should be made at specified intervals.

The investigators will make every effort to preserve the study subjects' confidentiality. No one other than study personnel will have access to the study participants' records. Precautions will be taken to ensure that the records are in a locked cabinet. Records will not have the subjects' names listed on them, but instead an identification number will be assign to each subject, which only the investigators and study coordinators will be able to link to subject names. The subjects will also not be identified in any reports of this study.

Research material will only be obtained from subjects who consent to participate. The data obtained are specifically for research purposes and will be maintained in a research study file, separate from the subject's medical record. Hard copies of the research records will be stored in a locked filing cabinet and electronic copies will be maintained on a secure server. Subjects will be assigned a unique identification number. This identification number will be linkable to the subject's identity via a database. The database will be stored on a secure computer server and will only be accessible by the study coordinators and the investigator.

10.1.3 FUTURE USE OF STORED SPECIMENS AND DATA

Not applicable.

10.1.4 KEY ROLES AND STUDY GOVERNANCE

Principal Investigator	Medical Monitor
Kevin C. Chung, MD, MS<br>Charles B.G. de Nancrede Professor in Surgery<br>Assistant Dean for Faculty Affairs (Instructional Track)	Not applicable.
University of Michigan Medical School	
Section of Plastic Surgery<br>Department of Surgery<br>University of Michigan Health System<br>1500 E. Medical Center Dr.<br>2130 Taubman Center, SPC 5340<br>Ann Arbor, MI 48109-5340	
734-615-3435	
kecchung@umich.edu	

10.1.5 SAFETY OVERSIGHT

10.1.5.1 TRAINING PLAN

A Consensus Meeting was held July 11th, 2009 at the Coordinating Center in Ann Arbor, MI. The goal of this meeting was to finalize the study protocol. Prior to this meeting the pertinent sections of the MOOP were distributed to the Clinical Sites for comments. These comments were incorporated and during the Consensus Meeting the protocol and MOOP were reviewed and final consensus was reached. Following the Consensus Meeting, the PI at each Clinical Site assigned a head Research Coordinator. This person was responsible for familiarizing themselves, the hand therapist(s) and any other research personnel with the study protocol.

A Kickoff Meeting was held August 27th, 2011 near the Coordinating Center in Romulus, MI. The goal of this meeting was to familiarize PIs and Research Coordinators from each site with the study protocol and procedures. PIs reached consensus on surgical techniques and inclusion/exclusion criteria. Research Coordinators went over patient recruitment, randomization, follow-up, data entry and adverse event reporting.

NIH policy requires that all PIs maintain certification in the Protection of Human Research Participants. Proof of certification is required to be provided to the University of Michigan upon request. It is the responsibility of each site to ensure that necessary personnel maintain certification.

All research personnel involved in any way with this project will have completed training in the protection of human research participants, per the guidelines issued by the U.S. Department of Health and Human Services, Office for Human Research Protection

10.1.5.2 MONITORING

In this study, the clinical sites will:

- Produce administrative reports that describe study progress to date, summarizing participant status (numbers screened, enrolled, completed, withdrawn, and discontinued treatment)
- Prepare other reports that list adverse events, serious adverse events, deaths, and disease or treatment specific events required for monitoring body review in order to ensure good clinical care and identify any emerging trends.
- Collect Serious Adverse Events from Performance Sites and report to the University of Michigan IRB
- Monitor data quality and protocol deviations through protocol deviation log.

Data Quality Review

- Coordinating Center will request all surveys and data entry forms for the first two months following the start of recruitment. Personnel at the Coordinating Center will double-enter these forms to confirm accurate data entry
- If a Performance Site is demonstrating accurate surveys and data entry forms will be requested for every tenth participant enrollment or follow-up visit.
  If a Performance Site is not demonstrating accurate data entry, corrective measures will be taken, including data entry training and site visits

10.1.6 *DATA HANDLING AND RECORD KEEPING*

10.1.6.1 DATA COLLECTION AND MANAGEMENT RESPONSIBILITIES

10.1.6.2 DATA COLLECTION FORMS

From each participant, data forms such as, Participant Demographic Questionnaire, RAPA, SF-36, MHQ, Hand Function Data form will be collected by the study coordinator at every visit. The surgeon will complete the Surgery Data form after surgery and complication checklist at every visit after surgery and these forms will also be collected by study coordinator.

The investigators will make every effort to preserve the study subjects' confidentiality. No one other than study personnel will have access to the study participants' records. Precautions will be taken to ensure that the records are in a locked cabinet. Records will not have the subjects' names listed on them, but instead an identification number will be assign to each subject, which only the investigators and study coordinators will be able to link to subject names. The subjects will also not be identified in any reports of this study.

To ensure compliance with the protocol, the Coordinating Center will create a MOP that is identical for each site. The MOP will be finalized after the study planning period. The MOP will contain instructions on how to complete all aspects of the study including randomization, measurements, data entry, and a copy of all study forms.

Data collection forms:

- Adverse Event Reporting form – Consists of the AE's with details pertaining to date of event, type of event, related or not, action taken and event outcome.
- Comorbidity Checklist – Questionnaire filled out by the participant at enrollment about their existing medical illness.
- Distal Radius Fracture Complication Checklist and Score Sheet- Form filled out by surgeon (PI) after surgery and every follow up visit, has information on complications developed such as nerve or bone/joint or tendon complications after surgery.
- General Health Questionnaire- information on general health in between follow up visits.
- Hand Edema Form – Measure of fingertip to palmar crease distance.
- Hand Function and Range of Motion data sheet- Measures grip, pinch and wrist range of motion of the injured arm and the normal arm.
- Hand Therapy Data Sheet – Information on type of therapy completed, number of visits, and when therapy was terminated.
- Inclusion/Exclusion Criteria – Includes fracture type and the detailed criteria for eligibility.
- Michigan Hand Outcomes Questionnaire - MHQ is a hand-specific outcomes instrument which measures the health outcomes of patients with chronic hand conditions
- Participant Demographic Questionnaire – Information on participant's gender, racial background, education etc
- Protocol Deviation log – consists of protocol deviation code and the date deviation occurred.
- Rapid Assessment of Physical Activity- RAPA is a questionnaire about the amount and intensity of physical activity patients usually do
- Screening Log – Consists of date of screening, patient demographic information, eligibility status and participation status.
- Serious Adverse Event Reporting form- Consists of the SAE's with details pertaining to date of event, type of event, category, related or not, relationship to research, action taken and event outcome
- SF-36 - survey of patients' health to assess their health status.
- Surgery Data Form- Consists of the procedure performed, prophylactic antibiotics used, DVT prophylaxis used, tourniquet time and brand and type of implant.

10.1.6.3 DATA MANAGEMENT

Clinical sites

Data will be double entered into the web-based data entry system by two separate individuals at each study site. The data entry system employs an "alert" system if out-of-range data are entered into the fields. After data have been entered into the data entry system, original hard

copies of all data will be mailed to the Coordinating Center once a month. Digital copies of x-rays (labeled with the patient's identification number and date taken) will also be mailed on CD.

Coordinating Center

The Coordinating Center will randomly spot-check the data entry for accuracy. Data can only be edited and downloaded from the web-based system by the Coordinating Center.
Coordinating Center will request all surveys and data entry forms for the first two months following the start of recruitment. Personnel at the Coordinating Center will double-enter these forms to confirm accurate data entry

10.1.6.4 STUDY RECORDS RETENTION

Each site will retain copies of the informed consent document, data collection forms, as well as maintaining an enrollment log and a database of their own participant contact and study information.

- Preparing and sending required reports to the coordinating center

  Clinical Sites will mail the original copies of consent forms and mail or fax data collection forms, as well as copies of the screening log, to the Coordinating Center on a monthly basis.
- Assuring IRB review and approval

  Clinical Sites will ensure that the project receives and maintains approval with their IRB. If IRB approval lapses or is withdrawn, Clinical Sites should inform the Coordinating Center as soon as possible.
- Communicating questions, concerns, and/or observations to the Coordinating Center

  Clinical Sites will address questions and concerns to the Coordinating Center as soon as possible, either by email or phone, depending on the urgency of the situation. Clinical Sites should keep a written record of all communications with the Coordinating Center. Observations and suggestions may be made to the Coordinating Center at any time.

10.1.7 PROTOCOL DEVIATIONS

Protocol deviations include, but are not limited to:

- Failure to obtain Informed Consent per study, institution or NIH standards
- Randomization of an ineligible participant
- Failure to keep IRB approval up to date
- Failure to report Adverse Events per institutional standards
- Failure to report Serious Adverse Events per study standards
- Wrong treatment administered to participant
- Participant follow-up occurs outside of specified time window
- Missing data or data forms
- Missing radiology data
- Data not transmitted to Coordinating Center on specified schedule

- Participant Cross-over

The Protocol Deviation log will be used by research personnel at each site to track any of the listed violations. Any such violation must be reported to the Coordinating Center within 24 hours of occurrence or as soon as it is discovered. Any such violations will then be reported to the DSMB by the Coordinating Center.

Every attempt to resolve any violation will be made as soon as it is discovered (e.g. if Informed Consent was not obtained prior to randomization, it should be obtained prior to surgery). This is the responsibility PI where the violation occurred.

Participant Cross-over

Participant cross-over, for any reason, should be reported to the Coordinating Center in the same manner. The Protocol Deviation log (Section Q2) should be completed and the Coordinating Center should be notified within 24 hours.

10.1.8 PUBLICATION AND DATA SHARING POLICY

Currently, we plan to present and publish data at the end of the third, fourth and fifth year. Authorship will collectively be referred to as “Wrist and Radius Injury Surgical Trial (WRIST).”

This study will be conducted in accordance with the following publication and data sharing policies and regulations:

National Institutes of Health (NIH) Public Access Policy, which ensures that the public has access to the published results of NIH funded research. It requires scientists to submit final peer-reviewed journal manuscripts that arise from NIH funds to the digital archive PubMed Central upon acceptance for publication.

This study will comply with the NIH Data Sharing Policy and Policy on the Dissemination of NIH-Funded Clinical Trial Information and the Clinical Trials Registration and Results Information Submission rule. As such, this trial will be registered at ClinicalTrials.gov, and results information from this trial will be submitted to ClinicalTrials.gov. In addition, every attempt will be made to publish results in peer-reviewed journals. Data from this study may be requested from other researchers x years after the completion of the primary endpoint by contacting <specify person or awardee institution, or name of data repository>.

10.2 ABBREVIATIONS

The list below includes abbreviations utilized in this template. However, this list should be customized for each protocol (i.e., abbreviations not used should be removed and new abbreviations used should be added to this list).

AE	Adverse Event
ANCOVA	Analysis of Covariance
CFR	Code of Federal Regulations

CLIA	Clinical Laboratory Improvement Amendments
CMP	Clinical Monitoring Plan
COC	Certificate of Confidentiality
CONSORT	Consolidated Standards of Reporting Trials
CRF	Case Report Form
DCC	Data Coordinating Center
DHHS	Department of Health and Human Services
DSMB	Data Safety Monitoring Board
DRE	Disease-Related Event
EC	Ethics Committee
eCRF	Electronic Case Report Forms
FDA	Food and Drug Administration
FDAAA	Food and Drug Administration Amendments Act of 2007
FFR	Federal Financial Report
GCP	Good Clinical Practice
GLP	Good Laboratory Practices
GMP	Good Manufacturing Practices
GWAS	Genome-Wide Association Studies
HIPAA	Health Insurance Portability and Accountability Act
IB	Investigator's Brochure
ICH	International Conference on Harmonisation
ICMJE	International Committee of Medical Journal Editors
IDE	Investigational Device Exemption
IND	Investigational New Drug Application
IRB	Institutional Review Board
ISM	Independent Safety Monitor
ISO	International Organization for Standardization
ITT	Intention-To-Treat
LSMEANS	Least-squares Means
MedDRA	Medical Dictionary for Regulatory Activities
MOP	Manual of Procedures
MSDS	Material Safety Data Sheet
NCT	National Clinical Trial
NIH	National Institutes of Health
NIH IC	NIH Institute or Center
OHRP	Office for Human Research Protections
PI	Principal Investigator
QA	Quality Assurance
QC	Quality Control
SAE	Serious Adverse Event
SAP	Statistical Analysis Plan
SMC	Safety Monitoring Committee
SOA	Schedule of Activities
SOC	System Organ Class
SOP	Standard Operating Procedure
UP	Unanticipated Problem
US	United States

10.3 PROTOCOL AMENDMENT HISTORY

version	version date	approval date	summary of changes
1	11/29/11	08/17/12	n/a
2	2/4/13	03/19/13	Participating Study Sites: administrative updates – site study staff/administrative staff names, site addresses and/or phone numbers<br>Precis: removed screening from participant table; updated recruitment goal<br>Section 3: updated randomization procedure<br>Section 4: updated collected demographic characteristics; updated screen procedure; updated info about collection and retention of ICDs<br>Section 5.2: updated info about blinding<br>Section 6.2: removed screening from schedule of evaluations; updated screening procedure; moved a paragraph about consent to after screening<br>Section 9.2: updated recruitment goal<br>Section 13: updated info about authorship<br>Section 15: added screening event
3	05/31/13	06/23/13	Participating Study Sites: administrative updates – site study staff/administrative staff names, site addresses and/or phone numbers<br>Section 6: Change in inclusion/exclusion criteria - Addition of Chinese language to inclusion criteria. This was necessary as older patients at the Singapore site were often unable to complete study documents in English but many speak Chinese.
4	01/23/14	03/10/14	Participating Study Sites: administrative updates – site study staff/administrative staff names, site addresses and/or phone numbers
5	10/13/14	11/11/14	Participating Study Sites: remove National University, Beth Israel, University of Washington; add University of Pittsburgh, Johns Hopkins University)
6	06/17/15	07/22/15	Participating Study Sites: administrative updates – site study staff/administrative staff names, site addresses and/or phone numbers<br>Section 6: Remove 2 week time frame from exclusion criteria
7	04/03/18	TBD	Correcting formatting prior to manuscript submission; put in most current NIAMS template<br>Coordinating center: Study staff changes<br>Section 9.4.3: Revised Analysis of secondary end point, Aim 5<br>Section 9.4.9: Revised Exploratory Analysis Aim 5

11 REFERENCES

1. Cummings SR, Black DM, Rubin SM. Lifetime risks of hip, Colles', or vertebral fracture and coronary heart disease among white postmenopausal women. *Arch Intern Med.* 1989;149:2445-2448.

2. Ray NF, Chan JK, Thamer M, Melton LJI. Medical expenditures for the treatment of osteoporotic fractures in the United States in 1995: report from the National Osteoporosis Foundation. *J Bone Miner Res.* 1997;12:24-35.

3. Burge RT, Dawson-Hughes B, King AB, Soloman DH, Tosteson A, Wong JB. Incidence and Economic Burden of Osteoporotic Fractures in the United States, 2005-2025. Paper presented at: National Osteoporosis Foundation - The Sixth International Symposium on Osteoporosis: Current Status and Future Directions.; April 7, 2005.

4. Jupiter JB, Ring D, Weitzel PP. Surgical treatment of redisplaced fractures of the distal radius in patients older than 60 years. *J Hand Surg.* 2002;27A:714-723.

5. Orbay JL, Fernandez DL. Volar fixed-angel plate fixation for unstable distal radius fractures in the elderly patient. *J Hand Surg.* 2004;29A:96-102.

6. Orbay JL, Fernandez DL. Volar fixation for dorsally displaced fractures of the distal radius: a preliminary report. *J Hand Surg.* 2002;27A(2):205-215.

7. Drobetz H, Kutscha-Lissberg E. Osteosynthesis of distal radius fractures with a volar locking screw plate system. *Int Orthop.* 2003;27:1-6.

8. Howard PW, Stewart HD, Hind RE, Burke FD. External fixation or plaster for severely displaced comminuted Colles' fractures? A prospective study of anatomical and functional results. *J Bone Joint Surg Br.* Jan 1989;71(1):68-73.

9. Horne JG, Devane P, Purdie G. A prospective randomized trial of external fixation and plaster cast immobilization in the treatment of distal radial fractures. *J Orthop Trauma.* 1990;4(1):30-34.

10. Leung F, Tu YK, Chew WY, Chow SP. Comparison of external and percutaneous pin fixation with plate fixation for intra-articular distal radial fractures. A randomized study. *J Bone Joint Surg Am.* Jan 2008;90(1):16-22.

11. Azzopardi T, Ehrendorfer S, Coulton T, Abela M. Unstable extra-articular fractures of the distal radius. *J Bone Joint Surg Br.* 2005;87(6):837-840.

12. Grewal R, Perey B, Wilmink M, Stothers K. A randomized prospective study on the treatment of intra-articular distal radius fractures: open reduction and internal fixation with dorsal plating verses mini open reduction, percutaneous fixation and external fixation. *J Hand Surg.* 2005;30A:764-772.

13. Wright TW, Hoorodyski M, Smith DW. Functional outcome of unstable radius fractures: ORIF with a volar fixed-angle tine plate versus external fixation. *J Hand Surg.* 2005;30A:289-299.

14. Westphal T, Piatek S, Schubert S, Winckler S. Outcome after surgery of distal radius fractures: no differences between external fixation and ORIF. *Arch Orthop Trauma Surg.* 2005;125:507-514.

15. Kapoor H, Agarwal A, Dhaon BK. Displaced intra-articular fractures of distal radius: a comparative evaluation of results following closed reduction, external fixation and open reduction with internal fixation. *Injury.* 2000;31(2):75-79.

16. Kreder HJ, Agel J, McKee MD, Schemitsch EH, Stephen D, Hanel DP. A randomized, controlled trial of distal radius fractures with metaphyseal displacement but without joint incongruity: closed reduction and casting versus closed reduction, spanning external fixation, and optional percutaneous K-wires. *J Orthop Trauma.* 2006;20(2):115-121.

17. Kreder HJ, Hanel DP, Agel J, et al. Indirect reduction and percutaneous fixation versus open reduction and internal fixation for displaced intra-articular fractures of the distal radius: a randomized, controlled trial. *J Bone Joint Surg.* 2005;87B:829-836.

18. Solgaard S, Petersen VS. Epidemiology of distal radius fractures. *Acta Orthop Scand.* 1985;56:391-393.

19. Singer BR, McLauchlan GJ, Robinson CM, Christie J. Epidemiology of fractures in 15,000 adults: the influence of age and gender. *J Bone Joint Surg.* 1998;80B:243-248.

20. O'Neill TW, Cooper C, Finn JD, et al. Incidence of distal forearm fracture in British men and women. *Osteoporos Int.* 2001;12:555-558.

21. Owen RA, Melton LJI. Incidence of Colles' fracture in a North American community. *Am J Public Health.* 1982;72:605-607.

22. Ring D, Jupiter JB. Treatment of osteoporotic distal radius fractures. *Osteoporos Int.* 2005;16:S80-84.

23. US Census Bureau PD. July 1, 2006 Population Estimates. 2007. Accessed May 29, 2007.

24. Vogt MT, Cauley JA, Tomaino MM, Stone K, Williams JR, Herndon JH. Distal radius fractures in older women: A 10-year follow-up study of descriptive characteristics and risk factors: The study of osteoporotic fractures. *J Am Geriatrics Society.* 2001;50:97-103.

25. MacDermid JC, Roth JH, Richards RS. Pain and disability reported in the year following a distal radius fracture: a cohort study. *BMC Musculoskelet Dis.* 2003;4:24.

26. Morris NS. Distal radius fracture in adults: self-reported physical functioning, role functioning, and meaning of injury. *Orthop Nurs.* 2000;19(4):37-48.

27. Madhok R, Green S. Longer term functional outcome and societal implications of upper limb fractures in the elderly. *J Roy Soc Health.* 1993;113(4):179-180.

28. Bialocerkowski AE. Difficulties associated with wrist disorders--a qualitative study. *Clin Rehabil.* 2002;16(4):429-440.

29. Chung KC, Burns PB. A guide to planning and executing a surgical randomized controlled trial. *J Hand Surg.* 2008;33A(3):407-412.

30. Kotsis SV, Lau FH, Chung KC. Responsiveness of the Michigan Hand Outcomes Questionnaire and physical measurements in outcome studies of distal radius fracture treatment. *J Hand Surg.* 2007;32A(1):84-90.

31. Chung KC. Michigan Hand Outcomes Questionnaire. 2008; *http://sitemaker.umich.edu/mhq/overview*. Accessed January 8, 2008.

32. Mahoney EM, Jurkovitz CT, Chu H, et al. Cost and cost-effectiveness of an early invasive vs conservative strategy for the treatment of unstable angina and non-ST-segment elevation myocardial infarction. *JAMA.* 2002;288:1851-1858.

33. Chung KC, Wei FC. An outcome study of thumb reconstruction using microvascular toe transfer. *J Hand Surg.* 2000;25A(4):651-658.

34. Chung KC, Kotsis SV. Outcomes of multiple microvascular toe transfers for reconstruction in 2 patients with digitless hands: 2- and 4-year follow-up case reports. *J Hand Surg.* 2002;27A(4):652-658.

35. Chung KC, Kotsis SV, Kim HM. A prospective outcomes study of Swanson metacarpophalangeal joint arthroplasty for the rheumatoid hand. *J Hand Surg.* 2004;29A(4):646-653.

36. Chung KC, Pillsbury MS, Walters MR, Hayward RA. Reliability and validity testing of the Michigan Hand Outcomes Questionnaire. *J Hand Surg.* 1998;23A(4):575-587.

37. Dias JJ, Burke FD, Wildin CJ, Heras-Palou C, Bradley MJ. Carpal tunnel syndrome and work. *J Hand Surg.* 2004;29B(4):329-333.

38. Mandl LA, Galvin DH, Bosch JP, et al. Metacarpophalangeal arthroplasty in rheumatoid arthritis: what determines satisfaction with surgery? *J Rheumatol.* 2002;29:2488-2491.

39. Kotsis SV, Chung KC. Responsiveness of the Michigan Hand Outcomes Questionnaire and the Disabilities of the Arm, Shoulder and Hand questionnaire in carpal tunnel surgery. *J Hand Surg.* 2005;30A(1):81-86.

40. Chung KC, Watt AJ, Kotsis SV, Margaliot Z, Haase SC, Kim HM. Treatment of unstable distal radial fractures with the volar locking plating system. *J Bone Joint Surg Am.* 2006;88(12):2687-2694.

41. Chung KC, Kotsis SV, Kim HM. Predictors of functional outcomes after surgical treatment of distal radius fractures. *J Hand Surg.* 2007;32A(1):76-83.

42. Chung KC, Squitiere L, Kim HM. A comparative outcomes study of using the volar locking plating system for distal radius fractures in both young and elderly adults. *J Hand Surg* 2008;33A:809-819.

43. Chung KC, Burns PB, Davis Sears E. Outcomes research in hand surgery: where have we been and where should we go? *J Hand Surg.* 2006;31A(8):1373-1379.

44. Chung KC, Walters MR, Greenfield ML, Chernew ME. Endoscopic versus open carpal tunnel release: a cost-effectiveness analysis. *Plast Reconstr Surg.* 1998;102(4):1089-1099.

45. Davis EN, Chung KC, Kotsis SV, Lau FH, Vijan S. A cost/utility analysis of open reduction and internal fixation versus cast immobilization for acute nondisplaced mid-waist scaphoid fractures. *Plast Reconstr Surg.* 2006;117(4):1223-1235.

46. Guyatt GH, Meade MO, Jaeschke RJ, Cook DJ, Haynes RB. Practitioners of evidence-based care. *BMJ.* 2000;320:945-955.

47. Amadio PC, Higgs P, Keith M. Prospective comparative trials in the Journal of Hand Surgery. *J Hand Surg.* 1996;21A:925-929.

48. Chung KC, Kalliainen LK, Hayward RA. Type II (beta) errors in the hand literature: the importance of power. *J Hand Surg.* 1998;23A(1):20-25.

49. Chung KC, Kalliainen LK, Spilson SV, Walters MR, Kim HM. The prevalence of negative studies with inadequate statistical power: an analysis of the plastic surgery literature. *Plast Reconstr Surg.* 2002;109(1):1-6.

50. *The outcome of outcomes research at AHCPR: final report.* Rockville, MD999.

51. Chung KC. Commentary: the quality of reporting and outcome measures in randomized clinical trials related to upper-extremity disorders. *J Hand Surg.* 2004;29A:735-737.

52. Chung KC, Shauver MJ, Birkmeyer JD. Trends in the United States in the treatment of distal radial fractures in the elderly. *J Bone Joint Surg Am.* Aug 2009;91(8):1868-1873.

53. Arora R, Gabl M, Gschwentner M, Deml C, Krappinger D, Lutz M. A comparative study of clinical and radiologic outcomes of unstable colles type distal radius fractures in patients older than 70 years: nonoperative treatment versus volar locking plating. *J Orthop Trauma.* 2009;23(4):237-242.

54. Fanuele J, Koval KJ, Lurie J, Zhou W, Tosteson A, Ring D. Distal radial fracture treatment: what you get may depend on your age and address. *J Bone Joint Surg Am.* 2009;91(6):1313-1319.

55. Synn AJ, Makhni EC, Makhni MC, Rozental TD, Day CS. Distal radius fractures in older patients: is anatomic reduction necessary? *Clin Orthop Relat Res.* 2009;467(6):1612-1620.

56. Shauver MJ, Chung KC. The minimal clinically important difference of the Michigan hand outcomes questionnaire. *J Hand Surg Am.* 2009;34(3):509-514.

57. Diggle P, Heagerty P, Liang K-Y, Zeger S. *Analysis of Longitudinal Data*. 2nd ed. New York: Oxford University Press; 2002.

58. Laird NM, Ware JH. Random-effects models for longitudinal data. *Biometrics.* 1982 1982;38:963-974.

59. Wei DH, Raizman NM, Bottino CJ, Jobin CM, Strauch RJ, Rosenwasser MP. Unstable distal radial fractures treated with external fixation, a radial column plate, or a volar plate. A prospective randomized trial. *J Bone Joint Surg Am.* 2009;91(7):1568-1577.

60. Grewal R, MacDermid JC. The risk of adverse outcomes in extra-articular distal radius fractures is increased with malalignment in patients of all ages but mitigated in older patients. *J Hand Surg [Am].* 2007;32(7):962-970.

61. Simic PM, Weiland AJ. Fractures of the distal aspect of the radius: changes in treatment over the past two decades. *Instr Course Lect.* 2003;52:185-195.

62. Raghunathan TE, Solenberger PW, Van Hoewyk J. IVEware: Imputation and Variance Estimation Software 2007; http://www.isr.umich.edu/src/smp/ive/.

63. Little RJA, Rubin DB. *Statistical Analysis with Missing Data.* Vol New York: Wiley; 1987.

64. Kim HM, Lagakos SW. Assessing drug compliance using longitudinal marker data, with application to AIDS. *Stat Med.* 1994;13(19-20):2141-2153.

65. Kreder HJ, Hanel DP, McKee M, Jupiter J, McGillivary G, Swiontkowski MF. Consistency of AO fracture classification for the distal radius. *J Bone Joint Surg Br.* 1996;78(5):726-731.

66. Beumer A, McQueen MM. Fractures of the distal radius in low-demand elderly patients: closed reduction of no value in 53 of 60 wrists. *Acta Orthop Scand.* 2003;74:98-100.

67. Young BT, Rayan GM. Outcome following nonoperative treatment of displaced distal radius fractures in low-demand patients older than 60 years. *J Hand Surg.* 2000;25A:19-28.

68. Anzarut A, Johnson JA, Rowe BH, Lambert RGW, Blitz S, Majumdar SR. Radiologic and patient-reported functional outcomes in an elderly cohort with conservatively treated fractures. *J Hand Surg.* 2004;29A:1121-1127.

69. Hernan MA, Brumback BA, Robins JM. Estimating the causal effect of zidovudine on CD4 count with a marginal structural model for repeated measures. *Stat Med.* 2002;21(12):1689-1709.

70. Siegel JE, Weinstein MC, Russell LB, Gold MR. Recommendations for reporting cost-effectiveness analyses. Panel on Cost-Effectiveness in Health and Medicine. *JAMA.* 1996;276:1339-1341.

71. Russell LB, Gold MR, Siegel JE, Daniels N, Weinstein MC. The role of cost-effectiveness analysis in health and medicine. Panel on Cost-Effectiveness in Health and Medicine. *JAMA.*1996;276:1172-1177.
