# Supplementary material for: Comparison of 24-Month Outcomes After Treatment for Distal Radius Fracture: The WRIST Randomized Clinical Trial
Source: JAMA Netw Open. 2021 Jun 17;4(6):e2112710. doi: 10.1001/jamanetworkopen.2021.12710 (PMC12507456; doi:10.1001/jamanetworkopen.2021.12710)
Supplement: Supplement 2. — eTable 1. Baseline Demographic and Clinical Characteristics by Treatment Groups in Those Who Completed 24-Months Assessment eTable 2. Descriptive Statistics for Baseline Values of Outcomes by Treatment Groups eTable 3. Unadjusted Means (95% Confidence Intervals) for Primary and Secondary Outcomes at 12- and 24-Month by Treatment Groups eTable 4. Descriptive Statistics for Primary and Secondary Outcomes at 24 Months by Malunion Status eFigure 1. MHQ Summary Scores Over the 24 Months Follow-up Assessment Times by Treatment Groups Using Data From All Cases eFigure 2. MHQ Summary Scores by the Pattern of Missingness, Separately for Each Treatment Group [file jamanetwopen-e2112710-s002.pdf]

Supplemental Online Content

Chung KC, Kim HM, Malay S, Shauver MJ; WRIST Group. Comparison of 24-month outcomes after treatment for distal radius fracture: the WRIST randomized clinical trial. *JAMA Netw Open*. 2021;4(6):e2112710. doi:10.1001/jamanetworkopen.2021.12710

eTable 1. Baseline Demographic and Clinical Characteristics by Treatment Groups in Those Who Completed 24-Months Assessment

eTable 2. Descriptive Statistics for Baseline Values of Outcomes by Treatment Groups

eTable 3. Unadjusted Means (95% Confidence Intervals) for Primary and Secondary Outcomes at 12- and 24-Month by Treatment Groups

eTable 4. Descriptive Statistics for Primary and Secondary Outcomes at 24 Months by Malunion Status

eFigure 1. MHQ Summary Scores Over the 24 Months Follow-up Assessment Times by Treatment Groups Using Data From All Cases

eFigure 2. MHQ Summary Scores by the Pattern of Missingness, Separately for Each Treatment Group

This supplemental material has been provided by the authors to give readers additional information about their work.

© 2021 Chung KC et al. *JAMA Network Open.*

eTable 1. Baseline Demographic and Clinical Characteristics by Treatment Groups in Those Who Completed 24-Months Assessment

**Variables**	**VLPS (N=50)**	**EFP (N=47)**	**CRPP (N=41)**	**Casting (N=44)**
	n (%)	n (%)	n (%)	n (%)
Female	40 (80.0)	45 (95.7)	35 (85.4)	40 (90.9%)
**Age,** mean (SD)	67.1 (6.3)	69.7 (8.5)	69.7 (6.5)	74.3 (10.6)
**Race**				
Minority	4 (8.0)	9 (19.2)	4 (9.8)	4 (9.1)
White	44 (88.0)	38 (80.9)	37 (90.2)	40 (90.9)
Missing	2 (4.0)	0 (0)	0 (0)	0 (0%
**Education**				
≤High school diploma/GED	16 (32.0)	12 (25.5)	17 (41.5)	16 (36.4)
Vocational school/associate's degree/some college	14 (28.0)	14 (29.8)	13 (31.7)	11 (25.0)
Bachelor's degree+	18 (36.0)	20 (42.6)	10 (24.4)	16 (36.4)
Missing	2 (4.0)	1 (2.1)	1 (2.4)	1 (2.3)
**Household Income**				
<$20,000	9 (18.0)	10 (21.3)	7 (17.1)	9 (20.5)
$20,000 - $39,000	8 (16.0)	8 (17.0)	8 (19.5)	13 (29.6)
$40,000 - $59,999	11 (22.0)	12 (25.5)	4 (9.8)	6 (13.6)
$60,000+	17 (34.0)	13 (27.7)	17 (41.5)	10 (22.7)
missing	5 (10.0)	4 (8.5)	5 (12.2)	6 (13.6)
**Functional Status – pre-injury**				
Sedentary	4 (8.0)	5 (10.6)	2 (4.9)	2 (4.6)
Under-active	20 (40.0)	21 (44.7)	20 (48.8)	25 (56.8)
Active	25 (50.0)	21 (44.7)	19 (46.3)	17 (38.6)
Missing	1 (2.0)	0 (0)	0 (0)	0 (0)
**Smoking status**				
Never	23 (46.0)	31 (66.0)	24 (58.5)	24 (54.6)
Former, <10 years	22 (44.0)	13 (27.7)	15 (36.6)	19 (43.2)
Current	4 (8.0)	3 (6.4)	2 (4.9)	1 (2.3)
missing	1 (2.0)	0 (0)	0 (0)	0 (0)
**No. comorbidities,** mean (SD)	3.1 (2.1)	3.5 (2.3)	3.4 (2.1)	3.5 (2.5)
**AO class**				
A1	0 (0)	1 (2.1)	0 (0)	1 (2.3)
A2	25 (50.0)	20 (42.6)	20 (48.8)	16 (36.4)
A3	3 (6.0)	8 (17.0)	5 (12.2)	9 (20.5)
C1	3 (6.0)	0 (0.0)	2 (4.9)	5 (11.4)
C2	15 (30.0)	15 (31.9)	12 (29.3)	9 (20.5)
C3	0 (0.0)	1 (2.1)	0 (0)	1 (2.3)
Missing	4 (8.0)	2 (4.3)	2 (4.9)	3 (6.8)
**Ulnar styloid fracture**				
Yes	23 (46.0)	26 (55.3)	16 (39.0)	18 (40.9)
No	24 (48.0)	19 (40.4)	23 (56.1)	22 (50.0)
Missing	3 (6.0)	2 (4.3)	2 (4.9)	4 (9.1)

© 2021 Chung KC et al. *JAMA Network Open.*

Note: No baseline variables showed differences (all $p>0.05$) across treatment groups, except age ($p<0.001$).

eTable 2. Descriptive Statistics for Baseline Values of Outcomes by Treatment Groups

Measures	VLPS (n=50)	EFP (n=47)	CRPP (n=41)	Casting (n=44)
MHQ Pain	70 (63-76)	63 (57-70)	62 (55-69)	56 (51-60)
SF36 PCS	34 (31-36)	33 (30-35)	36 (33-39)	35 (33-37)
SF36 MCS	48 (45-52)	50 (46-53)	51 (48-55)	49 (47-52)

Abbreviations: MHQ: The Michigan Hand Outcomes Questionnaire; SF-36 PCS: Short Form Physical Component Score, SF-36 MCS: Short Form Mental Component Score
Cell values are crude mean (95% confidence interval).
Note: For comparisons across treatment groups, only pain was significant ($p=0.005$).

© 2021 Chung KC et al. *JAMA Network Open.*

eTable 3. Unadjusted Means (95% Confidence Intervals) for Primary and Secondary Outcomes at 12- and 24-Month by Treatment Groups

Measures	Month	VLPS (N=50)	EFP (N=47)	CRPP (N=41)	Casting (N=44)	p-value*
**MHQ**						
Summary	24	88 (83-92)	83 (78-88)	85 (79-90)	85 (79-90)	0.81
	12	83 (78-88)	81 (76-86)	84 (80-89)	82 (77-87)	
Function	24	87 (82-92)^	83 (77-89)^	82 (77-88)	78 (72-85)	0.11
	12	79 (73-85)	77 (72-82)	78 (72-84)	76 (70-81)	
ADLs	24	91 (87-96)	85 (79-91)	89 (84-94)	85 (79-91)	0.86
	12	84 (79-90)	83 (77-88)	88 (84-92)	85 (79-91)	
Work	24	87 (80-93)	85 (79-91)	88 (82-94)	86 (81-92)	0.58
	12	79 (73-86)	82 (75-89)	84 (78-90)	83 (77-88)	
Pain	24	11 (5-17)^	14 (8-19)^	14 (8-20)	15 (9-22)	0.20
	12	20 (14-26)	21 (15-27)	20 (14-26)	16 (11-21)	
Aesthetics	24	86 (80-93)	84 (77-90)	87 (80-93)	89 (83-94)	0.53
	12	86 (81-91)	83 (77-89)	85 (79-91)	83 (78-88)	
Satisfaction	24	87 (80-94)	83 (76-90)	79 (70-89)	79 (70-88)	0.94
	12	79 (72-87)	76 (69-83)	80 (73-88)	78 (72-85)	
**SF-36**						
PCS	24	46 (43-49)	46 (43-49)	49 (45-52)	46 (42-49)	0.90
	12	46 (43-49)	46 (43-49)	48 (44-51)	47 (44-50)	
MCS	24	56 (53-58)	54 (51-57)	54 (51-57)	53 (50-56)^	0.08
	12	54 (51-56)	53 (50-57)	55 (53-57)	57 (55-59)	
**Functional measures, % of uninjured hand/wrist**						
Grip strength	24	90 (83-97)	81 (74-88)^	89 (84-95)^	85 (78-92)^	0.007
	12	84 (79-90)	73 (65-80)	82 (77-86)	80 (75-84)	
Pinch strength	24	99 (94-105)^	92 (85-99)	99 (85-113)^	102 (91-113)¶	<0.001
	12	93 (87-98)	86 (82-90)	91 (86-95)	86 (82-90)	
Flexion	24	91 (85-96)	94 (85-102)^	89 (83-95)	83 (76-91)	0.28
	12	87 (82-91)	86 (80-91)	85 (78-92)	80 (74-87)	
Extension	24	95 (88-101)	90 (85-95)	94 (90-99)	94 (87-101)	0.54
	12	96 (96-102)	87 (81-92)	90 (85-95)	97 (91-102)	
Ulnar Deviation	24	98 (91-105)	91 (83-100)	102 (90-115)	97 (87-107)	0.22
	12	91 (84-99)	84 (79-89)	92 (83-101)	89 (70-107)	
Radial Deviation	24	99 (87-112)	92 (83-100)	108 (92-124)^	115 (96-133)	0.03
	12	92 (82-102)	100 (88-113)	89 (77-101)	115 (102-129)	

© 2021 Chung KC et al. *JAMA Network Open.*

Pronation	24	99 (98-100)	99 (97-101)	99 (97-101)	97 (90-104)	0.80
	12	99 (98-100)	96 (94-98)	99 (98-100)	96 (92-100)	
Supination	24	104 (94-114)	95 (93-98)	94 (90-98)	94 (90-99)	0.63
	12	99 (93-105)	94 (90-99)	100 (94-106)	95 (92-97)	

*From testing for the difference in changes in means from between month 12 and 24 within each of the four treatment groups jointly using a linear mixed-effects model with data at all follow-up time from all four treatment groups adjusting for categorical time indicators, treatment group indicators, time by treatment group interactions. age, baseline pain score, current smoking status, activity level (RAPA), and race.

^, ¶ From testing significance of covariate adjusted changes between month 12 and 24 within each treatment group; ^ for p-value between 0.001 and 0.05 and ¶ for p-value <0.001.

© 2021 Chung KC et al. *JAMA Network Open.*

eTable 4. Descriptive Statistics for Primary and Secondary Outcomes at 24 Months by Malunion Status

	No malunion	Malunion	p-value*
**N**	**140**	**42**	
**MHQ, Crude mean (95% CI)**			
Summary	86 (83-89)	83 (77-89)	0.31
Function	84 (81-87)	78 (71-85)	0.08
ADLs	89 (86-92)	84 (78-90)	0.12
Work	88 (85-91)	81 (74-88)	0.06
Pain	13 (10-16)	15 (7-22)	0.61
Aesthetics	87 (84-90)	84 (77-91)	0.42
Satisfaction	83 (79-88)	80 (71-89)	0.49
**SF-36, Crude mean (95% CI)**			
PCS	46 (44-48)	47 (43-50)	0.93
MCS	55 (53-56)	52 (49-55)	0.15
**Functional measures, % of uninjured hand/wrist, Crude mean (95% CI)**			
Grip strength	87 (83-90)	85 (75-95)	0.72
Pinch strength	98 (93-103)	99 (86-113)	0.78
Flexion	91 (88-95)	83 (75-92)	0.06
Extension	93 (89-96)	95 (88-102)	0.45
Ulnar Deviation	99 (94-105)	89 (81-97)	0.08
Radial Deviation	103 (94-111)	104 (92-117)	0.84
Pronation	99 (97-101)	99 (96-102)	0.80
Supination	97 (93-101)	98 (96-101)	0.69
**Employment at 24 month, n (%)**			
Full-time	19 (13.6)	3 (7.1)	0.60
Part-time	19 (13.6)	3 (7.1)	
Retired	87 (62.1)	31 (73.8)	
Receiving disability	6 (4.3)	1 (2.4)	
Unemployed	4 (2.9)	2 (4.8)	
Missing	5 (3.6)	2 (4.8)	
**Physical Activity at 24 months, n (%)**			
Sedentary	11 (7.9)	3 (7.1)	0.65
Under-active	69 (49.3)	18 (42.9)	
Active	49 (35.0)	19 (45.2)	
missing	11 (7.9)	2(4.8)	

Abbreviations: MHQ=Michigan Hand Outcomes Questionnaire; ADL=activities of daily living; PCS=Physical Component Score; MCS=Mental Component Score

Note: All hand outcomes are specific to injured side (hand or wrist), except Work subdomain which has one score for both hands. Grip strength and key pinch strength are % injured hand out of uninjured hand, and for other functional measures are % injured wrist out of uninjured wrist.

*P-values are from testing for the difference between those with malunion vs. not using t-test for continuous variables and chi-square tests for categorical outcomes.

© 2021 Chung KC et al. *JAMA Network Open.*

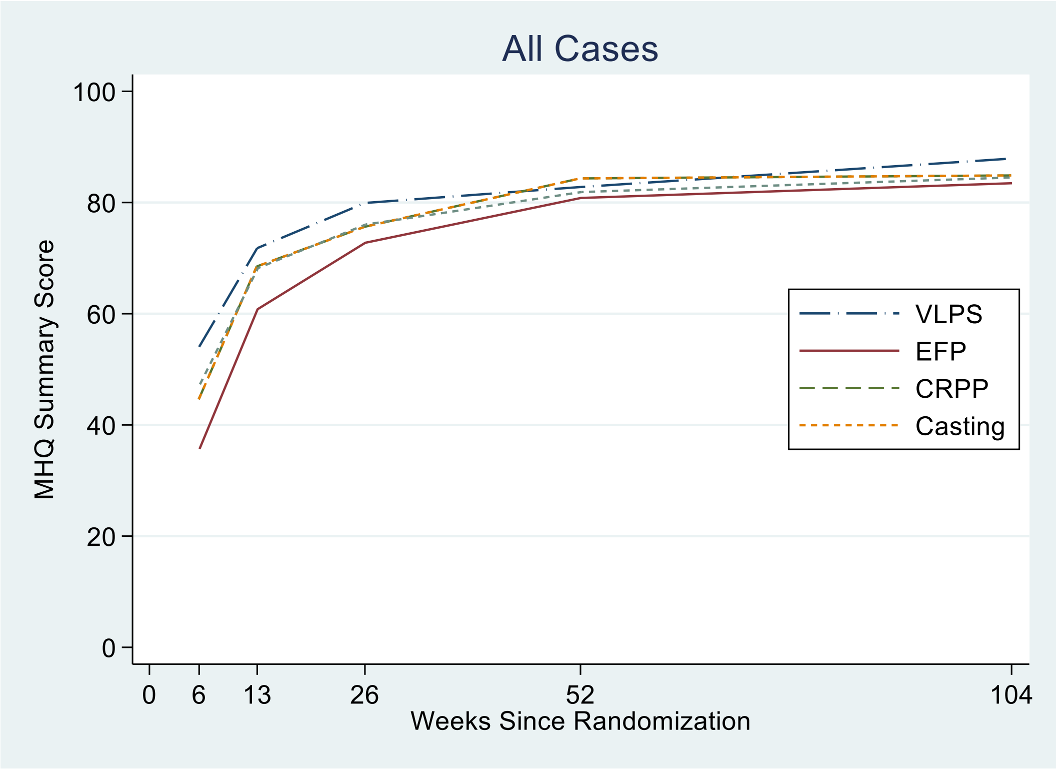

eFigure 1. MHQ Summary Scores Over the 24 Months Follow-up Assessment Times by Treatment Groups Using Data From All Cases

© 2021 Chung KC et al. *JAMA Network Open.*

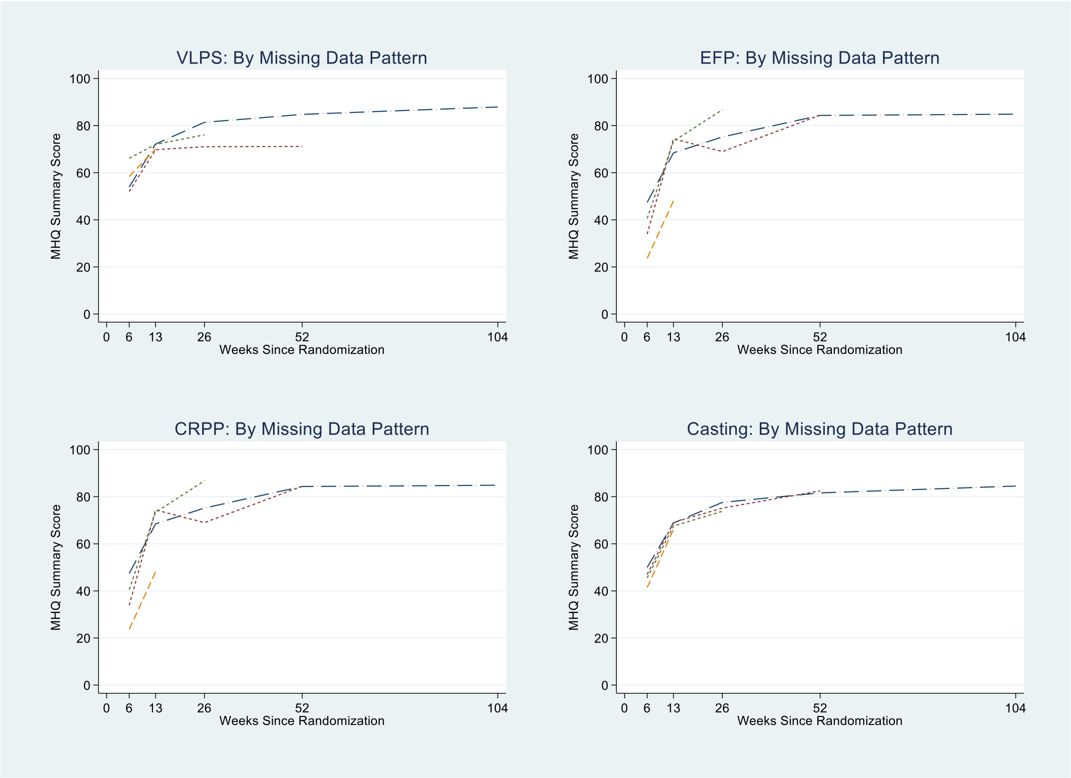

eFigure 2. MHQ Summary Scores by the Pattern of Missingness, Separately for Each Treatment Group

No notable difference in mean MHQ summary scores over time is seen by the missing data pattern across treatment groups.

© 2021 Chung KC et al. *JAMA Network Open.*
