# Supplementary material for: Comparison of 24-Month Outcomes After Treatment for Distal Radius Fracture: The WRIST Randomized Clinical Trial
Source: JAMA Netw Open. 2021 Jun 17;4(6):e2112710. doi: 10.1001/jamanetworkopen.2021.12710 (PMC12507456; doi:10.1001/jamanetworkopen.2021.12710)
Supplement: Supplement 3. — WRIST Group Members [file jamanetwopen-e2112710-s003.pdf]

Supplemental Online Content: Nonauthor Collaborators

*Indicates required information. Only first name, last name, and suffix will appear in PubMed.

*Group Name(s): WRIST (Wrist and Radius Surgery Injury Trial) Group							
***First Name and Middle Initial(s)**	***Last Name**	***Suffix (eg, Jr, III)**	Academic Degrees	Institution	Location (city, state/province, country)	Role or Contribution, eg, chair, principal investigator	Group (if more than 1 Group listed in the byline) and/or Subgroup (eg, Steering Committee)
Steven C	Haase		MD	Michigan Medicine	Ann Arbor, MI	Co-Investigator	
Jeffrey N	Lawton		MD	Michigan Medicine	Ann Arbor, MI	Co-Investigator	
John R	Lien		MD	Michigan Medicine	Ann Arbor, MI	Co-Investigator	
Adeyiza O	Momoh		MD	Michigan Medicine	Ann Arbor, MI	Co-Investigator	
Kagan	Ozer		MD	Michigan Medicine	Ann Arbor, MI	Co-Investigator	
Erika D	Sears		MD	Michigan Medicine	Ann Arbor, MI	Co-Investigator	
Jennifer F	Waljee		MD, MPH	Michigan Medicine	Ann Arbor, MI	Co-Investigator	
Matthew s	Brown		MD	Michigan Medicine	Ann Arbor, MI	Hand Fellow	
Hoyune E	Cho		MD	Michigan Medicine	Ann Arbor, MI	Fellow	
Brett F	MIchelotti		MD	Michigan Medicine	Ann Arbor, MI	Hand Fellow	
Tamara D	Rozental		MD	Beth Israel Deaconess Medical Ce	Boston, MA	Site Principal Investigator	
Paul T.	Appleton		MD	Beth Israel Deaconess Medical Ce	Boston, MA	Co-Investigator	
Edward K	Rodriguez		MD, PhD	Beth Israel Deaconess Medical Ce	Boston, MA	Co-Investigator	
Laura N.	Deschamps		DO	Beth Israel Deaconess Medical Ce	Boston, MA	Co-Investigator	
Lindsay	Mattfolk		BA	Beth Israel Deaconess Medical Ce	Boston, MA	Research coordinator	
Katiri	Wagner			Beth Israel Deaconess Medical Ce	Boston, MA	Research coordinator	
Philip	Blazar		MD	Brigham and Women's Hospital	Boston, MA	Site Principal Investigator	
Brandon E	Earp		MD	Brigham and Women's Hospital	Boston, MA	Co-Investigator	
Emerson	Floyd			Brigham and Women's Hospital	Boston, MA	Research coordinator	
Dexter L	Louie		BS	Brigham and Women's Hospital	Boston, MA	Research coordinator	
Fraser J.	Leversedge		MD	Duke Health	Durham, NC	Site Principal Investigator	
Marc J.	Richard		MD	Duke Health	Durham, NC	Co-Investigator	
David S	Ruch		MD	Duke Health	Durham, NC	Co-Investigator	
Suzanne	Finley			Duke Health	Durham, NC	Research coordinator	
Cameron	Howe			Duke Health	Durham, NC	Research coordinator	
Maria	Manson			Duke Health	Durham, NC	Research coordinator	
Janna	Whitfield		BS	Duke Health	Durham, NC	Research coordinator	

Supplemental Online Content: Nonauthor Collaborators

*Indicates required information. Only first name, last name, and suffix will appear in PubMed.

*First Name and Middle Initial(s)	*Last Name	*Suffix (eg, Jr, III)	Academic Degrees	Institution	Location (city, state/province, country)	Role or Contribution, eg, chair, principal investigator	Group (if more than 1 Group listed in the byline) and/or Subgroup (eg, Steering Committee)
Bertrand	Perey		MD	Fraser Health Authority	Vancouver, CA	Site Principal Investigator	
Kelly	Apostle		MD, FRCSC	Fraser Health Authority	Vancouver, CA	Co-Investigator	
Dory	Boyer		MD, FRCSC	Fraser Health Authority	Vancouver, CA	Co-Investigator	
Farhad	Moola		MD, FRCSC	Fraser Health Authority	Vancouver, CA	Co-Investigator	
Trevor	Stone		MD, FRCSC	Fraser Health Authority	Vancouver, CA	Co-Investigator	
Darous	Viskontas		MD, FRCSC	Fraser Health Authority	Vancouver, CA	Co-Investigator	
Mauri	Zomar			Fraser Health Authority	Vancouver, CA	Research coordinator	
Karyn	Moon			Fraser Health Authority	Vancouver, CA	Research coordinator	
Raely	Moon			Fraser Health Authority	Vancouver, CA	Research coordinator	
Loree K	Kalliainen		MD, MA	HealthPartners Institute for Educa	Bloomington, Minnesota	Site Principal Investigator	
Christina M	Ward		MD	HealthPartners Institute for Educa	Bloomington, Minnesota	Co-Investigator	
James W	Fletcher		MD	HealthPartners Institute for Educa	Bloomington, Minnesota	Co-Investigator	
Cherrie A	Heinrich		MD	HealthPartners Institute for Educa	Bloomington, Minnesota	Co-Investigator	
Katharine S	Pico		MD	HealthPartners Institute for Educa	Bloomington, Minnesota	Co-Investigator	
Ashish Y.	Mahajan		MD	HealthPartners Institute for Educa	Bloomington, Minnesota	Co-Investigator	
Brian W	Hill		MD	HealthPartners Institute for Educa	Bloomington, Minnesota	Co-Investigator	
Sandy	Vang		BS	HealthPartners Institute for Educa	Bloomington, Minnesota	Research coordinator	
Dawn M	Laporte		MD	Johns Hopkins Medicine	Baltimore, MD	Site Principal Investigator	
Erik A	Hasenboehler		MD	Johns Hopkins Medicine	Baltimore, MD	Co-Investigator	
Scott D	Lifchez		MD	Johns Hopkins Medicine	Baltimore, MD	Co-Investigator	
Greg M	Osgood		MD	Johns Hopkins Medicine	Baltimore, MD	Co-Investigator	
Babar	Shafiq		MD	Johns Hopkins Medicine	Baltimore, MD	Co-Investigator	
Jaimie T	Shores		MD	Johns Hopkins Medicine	Baltimore, MD	Co-Investigator	
Vaishali	Laljani			Johns Hopkins Medicine	Baltimore, MD	Research coordinator	
Brent H	Bamberger		DO	Kettering Health Network	Dayton, OH	Site Principal Investigator	
Timothy W	Harman		DO	Kettering Health Network	Dayton, OH	Co-Investigator	
David W	Martineau		MD	Kettering Health Network	Dayton, OH	Co-Investigator	

Supplemental Online Content: Nonauthor Collaborators

*Indicates required information. Only first name, last name, and suffix will appear in PubMed.

*First Name and Middle Initial(s)	*Last Name	*Suffix (eg, Jr, III)	Academic Degrees	Institution	Location (city, state/province, country)	Role or Contribution, eg, chair, principal investigator	Group (if more than 1 Group listed in the byline) and/or Subgroup (eg, Steering Committee)
Carla	Robinson		PA-C, MPAS	Kettering Health Network	Dayton, OH	Research coordinator	
Brandi	Palmer		MS, PC, CCRP	Kettering Health Network	Dayton, OH	Research coordinator	
Ruby	Grewal		MD	London Health Sciences Centre	London, CA	Site Principal Investigator	
Ken A	Faber		MD	London Health Sciences Centre	London, CA	Co-Investigator	
Joy C	MacDermid		PhD	London Health Sciences Centre	London, CA	Study Epidemiologist	
Kate	Kelly		MSc, MPH	London Health Sciences Centre	London, CA	Research coordinator	
Katrina	Munro			London Health Sciences Centre	London, CA	Research coordinator	
Joshua I	Vincent		PT, PhD	London Health Sciences Centre	London, CA	Research coordinator	
David	Ring		MD, PhD	Massachusetts General Hospital,	Boston, MA	Site Principal Investigator	
Jesse B	Jupiter		MD, MA	Massachusetts General Hospital,	Boston, MA	Co-Investigator	
Jilian S	Gruber		MD	Massachusetts General Hospital,	Boston, MA	Co-Investigator	
Abigail	Finger		BA	Massachusetts General Hospital,	Boston, MA	Research coordinator	
Rakesh K	Reddy			Massachusetts General Hospital,	Boston, MA	Research coordinator	
Taylor M	Pong			Massachusetts General Hospital,	Boston, MA	Research coordinator	
Emily	Thornton		BSc	Massachusetts General Hospital,	Boston, MA	Research coordinator	
David G	Dennison		MD	Mayo Clinic	Rochester, MN	Site Principal Investigator	
Sanjeev	Kakar		MD	Mayo Clinic	Rochester, MN	Co-Investigator	
Marco	Rizzo		MD	Mayo Clinic	Rochester, MN	Co-Investigator	
Alexander Y	Shin		MD	Mayo Clinic	Rochester, MN	Co-Investigator	
Tyson L	Scrabeck			Mayo Clinic	Rochester, MN	Research coordinator	
Kyle	Chepla		MD	The MetroHealth System	Cleveland OH	Site Principal Investigator	
Kevin	Malone		MD	The MetroHealth System	Cleveland OH	Co-Investigator	
Harry A	Hoyen		MD	The MetroHealth System	Cleveland OH	Co-Investigator	
Blaine Todd	Bafus		MD	The MetroHealth System	Cleveland OH	Co-Investigator	
Roderick B	Jordan		MD	The MetroHealth System	Cleveland OH	Co-Investigator	
Bram	Kaufman		MD	The MetroHealth System	Cleveland OH	Co-Investigator	
Ali	Totonchil		MD	The MetroHealth System	Cleveland OH	Co-Investigator	
Dana R	Hromyak		BS	The MetroHealth System	Cleveland OH	Research coordinator	

Supplemental Online Content: Nonauthor Collaborators

*Indicates required information. Only first name, last name, and suffix will appear in PubMed.

*First Name and Middle Initial(s)	*Last Name	*Suffix (eg, Jr, III)	Academic Degrees	Institution	Location (city, state/province, country)	Role or Contribution, eg, chair, principal investigator	Group (if more than 1 Group listed in the byline) and/or Subgroup (eg, Steering Committee)
Lisa	Humbert		RN	The MetroHealth System	Cleveland OH	Research coordinator	
Sandeep	Sebastin		MCh	National University of Singapore	Singapore	Site Principal Investigator	
Sally	Tay			National University of Singapore	Singapore	Research coordinator	
Kate W	Nellans		MD, MPH	Northwell Health	Newyok, NY	Site Principal Investigator	
Sara L	Merwin		MPH	Northwell Health	Newyok, NY	Research coordinator	
Ethan W	Blackburn		MD	Norton Healthcare	Louiville, KY	Site Principal Investigator	
Sandra J	Hanlin		APRN, NP-C	Norton Healthcare	Louiville, KY	Research coordinator	
Barbara	Patterson		CCRC	Norton Healthcare	Louiville, KY	Research coordinator	
Glenn R	Gaston		MD	OrthoCarolina Research Institute	Charlotte, NC	Site Principal Investigator	
Christopher R	Chadderdon		MD	OrthoCarolina Research Institute	Charlotte, NC	Co-Investigator	
Erika G	Gantt		MD	OrthoCarolina Research Institute	Charlotte, NC	Co-Investigator	
John S	Gaul		MD	OrthoCarolina Research Institute	Charlotte, NC	Co-Investigator	
Daniel R	Lewis		MD	OrthoCarolina Research Institute	Charlotte, NC	Co-Investigator	
Bryan J	Leffler		MD	OrthoCarolina Research Institute	Charlotte, NC	Co-Investigator	
Lois K	Osier		MD	OrthoCarolina Research Institute	Charlotte, NC	Co-Investigator	
Paul C	Perlik		MD	OrthoCarolina Research Institute	Charlotte, NC	Co-Investigator	
Alan W	Ward		MD	OrthoCarolina Research Institute	Charlotte, NC	Co-Investigator	
Tod A	Clark		MD, MSc, FRC	Pan Am Clinic/University of Manito	Winnipeg, CA	Site Principal Investigator	
Sheila	McRae		MSc, PhD	Pan Am Clinic/University of Manito	Winnipeg, CA	Research coordinator	
Jennifer M	Wolf		MD	University of Connecticut Health	Farmington, CT	Site Principal Investigator	
Craig M	Rodner		MD	University of Connecticut Health	Farmington, CT	Co-Investigator	
Katy	Coyle		RN	University of Connecticut Health	Farmington, CT	Research coordinator	
Thomas P	Lehman		MD	University of Oklahoma Medicine	Oklahoma City, OK	Site Principal Investigator	
Yuri C	Lansinger		MD	University of Oklahoma Medicine	Oklahoma City, OK	Co-Investigator	
Gavin D	O'Mahony		MD	University of Oklahoma Medicine	Oklahoma City, OK	Co-Investigator	
Kathy	Carl		BA, CCRP	University of Oklahoma Medicine	Oklahoma City, OK	Research coordinator	

Supplemental Online Content: Nonauthor Collaborators

*Indicates required information. Only first name, last name, and suffix will appear in PubMed.

***First Name and Middle Initial(s)**	***Last Name**	***Suffix (eg, Jr, III)**	Academic Degrees	Institution	Location (city, state/province, country)	Role or Contribution, eg, chair, principal investigator	Group (if more than 1 Group listed in the byline) and/or Subgroup (eg, Steering Committee)
Janet	Wells			University of Oklahoma Medicine	Oklahoma City, OK	Research coordinator	
David J	Bozentka		MD	University of Pennsylvania Health	Philadelphia, PA	Site Principal Investigator	
Scott L	Levin		MD	University of Pennsylvania Health	Philadelphia, PA	Co-Investigator	
David P	Steinberg		MD	University of Pennsylvania Health	Philadelphia, PA	Co-Investigator	
Robert	Goitz		MD	University of Pennsylvania Health	Philadelphia, PA	Co-Investigator	
Annamarie D	Horan		PhD	University of Pennsylvania Health	Philadelphia, PA	Co-Investigator	
Denise	Knox		BS	University of Pennsylvania Health	Philadelphia, PA	Research coordinator	
Kara	Napolitano		BS	University of Pennsylvania Health	Philadelphia, PA	Research coordinator	
Cathy A	Naccarelli			University of Pennsylvania Health	Philadelphia, PA	Research coordinator	
Joelle	Tighe			University of Pennsylvania Health	Philadelphia, PA	Research coordinator	
Warren C	Hammert		MD, DDS	University of Rochester	Rochester, NY	Site Principal Investigator	
Allison W	McIntyre		MPH	University of Rochester	Rochester, NY	Research coordinator	
Krista L	Noble			University of Rochester	Rochester, NY	Research coordinator	
Kaili	Waldrick			University of Rochester	Rochester, NY	Research coordinator	
Jeffrey B	Friedrich		MD	University of Washington Medicine	Seattle, WA	Site Principal Investigator	
David	Bowman			University of Washington Medicine	Seattle, WA	Research coordinator	
Angela	Wilson			University of Washington Medicine	Seattle, WA	Research coordinator	
Zhongyu	LI		MD, PhD	Wake Forest Baptist Health	Winstn-Salem, NC	Site Principal Investigator	
Andrew L	Koman		MD	Wake Forest Baptist Health	Winstn-Salem, NC	Co-Investigator	
Benjamin R	Graves		MD	Wake Forest Baptist Health	Winstn-Salem, NC	Co-Investigator	
Beth P	Smith		PhD	Wake Forest Baptist Health	Winstn-Salem, NC	Research coordinator	
Debra	Bullard			Wake Forest Baptist Health	Winstn-Salem, NC	Research coordinator	
