# Supplementary material for: Comparison of 24-Month Outcomes After Treatment for Distal Radius Fracture: The WRIST Randomized Clinical Trial
Source: JAMA Netw Open. 2021 Jun 17;4(6):e2112710. doi: 10.1001/jamanetworkopen.2021.12710 (PMC12507456; doi:10.1001/jamanetworkopen.2021.12710)
Supplement: Supplement 4. — Data Sharing Statement [file jamanetwopen-e2112710-s004.pdf]

Data Sharing Statement

Chung. Comparison of 24-Month Outcomes After Treatment for Distal Radius Fracture. *JAMA Netw Open*. Published June 17, 2021. doi:10.1001/jamanetworkopen.2021.12710

Data
Data available: No
